# More rTMS pulses or more sessions? The impact on treatment outcome for treatment resistant depression

**DOI:** 10.1111/acps.13768

**Published:** 2024-11-21

**Authors:** E. Oostra, P. Jazdzyk, V. Vis, I. Dalhuisen, A. W. Hoogendoorn, C. H. M. Planting, P. F. van Eijndhoven, Y. D. van der Werf, O. A. van den Heuvel, E. van Exel

**Affiliations:** ^1^ Amsterdam UMC, Dept. Psychiatry Vrije Universiteit Amsterdam Amsterdam Netherlands; ^2^ Amsterdam UMC, Dept Anatomy & Neuroscience Vrije Universiteit Amsterdam Amsterdam Netherlands; ^3^ GGZ inGeest Specialized Mental Health Care Amsterdam Netherlands; ^4^ Amsterdam Neuroscience, Mood, Anxiety, Psychosis, Sleep & Stress program Amsterdam Netherlands; ^5^ Second Department of Psychiatry Institute of Psychiatry and Neurology Warsaw Poland; ^6^ Chair and Department of Experimental and Clinical Physiology, Laboratory of Centre for Preclinical Research Medical University of Warsaw Warsaw Poland; ^7^ Department of Psychiatry Radboud University Medical Center Nijmegen HB Netherlands; ^8^ Donders Institute of Brain Cognition and Behavior Centre for Neuroscience Nijmegen HE Netherlands; ^9^ Amsterdam Neuroscience, Compulsivity Impulsivity Attention Amsterdam Netherlands

**Keywords:** meta‐analysis, meta‐regression, repetitive transcranial magnetic stimulation, treatment resistant depression

## Abstract

**Background:**

Repetitive transcranial magnetic stimulation (rTMS) is effective for treatment‐resistant depression (TRD). Optimal rTMS parameters remain unclear, especially whether number of sessions or amount of pulses contribute more to treatment outcome. We hypothesize that treatment outcome depends on the number of sessions rather than on the amount of pulses.

**Methods:**

We searched databases for randomized clinical trials (RCTs) on high‐frequent (HF) or low‐frequent (LF)‐rTMS targeting the left or right DLPFC for TRD. Treatment efficacy was measured using standardized mean difference (SMD), calculated from pre‐ and post‐treatment depression scores. Meta‐regressions were used to explore linear associations between SMD and rTMS pulses, pulses/session and sessions for HF and LF‐rTMS, separately for active and sham‐rTMS. If these variables showed no linear association with SMD, we divided the data into quartiles and explored subgroup SMDs.

**Results:**

Eighty‐seven RCTs were included: 67 studied HF‐rTMS, eleven studied LF‐rTMS, and nine studied both. No linear association was found between SMD and amount of pulses or pulses/session for HF and LF‐rTMS. Subgroup analyses showed the largest SMDs for 1200–1500 HF‐pulses/session and 360–450 LF‐pulses/session. The number of sessions was significantly associated with SMD for active HF (*β* = 0.09, *p* < 0.05) and LF‐rTMS (*β* = 0.06, *p* < 0.01). Thirty was the maximal number of sessions, in the included RCTs.

**Conclusion:**

More rTMS sessions, but not more pulses, were associated with improved treatment outcome, in both HF and LF‐rTMS. Our findings suggest that 1200–1500 HF‐pulses/session and 360–450 LF‐pulses/session are already sufficient, and that a treatment course should consist of least 30 sessions for higher chance of response.

**Summations**

- Increasing the amount of rTMS pulses does not increase the chances on treatment response.
- Receiving more sessions was significantly associated with higher treatment response.

**Limitations**

- The included studies showed high levels of heterogeneity.
- This study did not investigate the longitudinal contribution of number of sessions or rTMS pulses on treatment response.

## 1 INTRODUCTION

Treatment‐resistant depression (TRD) is a severe condition where patients suffering from a depressive episode show insufficient response to several evidence‐based treatment options such as antidepressant medication or psychotherapy.^1^ Patients with TRD are twice as likely to be hospitalized and show a higher suicide risk compared with individuals responding well to treatment for major depressive disorder.^2^, ^3^ Repetitive transcranial magnetic stimulation (rTMS) is an effective treatment in patients suffering from TRD.^4^, ^5^ However, response and remission rates vary widely; 39.5%–70% and 16.6%–76.9%, respectively.^6^ A possible explanation for the high variation in response and remission rates reported in published trials could be related to rTMS dosage, which could be conceptualized to depend on both amount of pulses and treatment sessions.

The U.S. Food and Drug Administration (FDA) cleared the first TMS machine for clinical use in treating TRD. Their labeled protocol in 2008 for high‐frequency (HF‐) rTMS only (not for low‐frequency (LF‐) rTMS), prescribed rTMS parameters including stimulation location (left dorsolateral prefrontal cortex; l‐DLPFC), stimulation intensity (120% of the motor threshold (MT)), stimulation frequency (10 Hz), pulses per session (3000 pulses/session), and a range of number of sessions (between 20 and 30 sessions).^7^, ^8^

In the past decades of rTMS research, several variations of the FDA‐approved protocol parameters have been investigated.^9^ Fitzgerald and colleagues^10^ investigated in their randomized controlled trial (RCT) the relationship between the total amount of pulses administered for both HF and LF‐rTMS protocols in TRD, differing between standard dose (HF: 2250 pulses/session, LF: 1200 pulses/session) and high dose (HF: 5625 pulses/session, LF: 3600 pulses/session). All four protocols consisted of 20 daily sessions in total. The protocols did not show a statistically significant difference in clinical response, suggesting that the amount of pulses administered during rTMS treatment has a limited effect on treatment outcome. On the other hand, Hutton and colleagues^11^ found in their naturalistic study using an extensive patient registry of 7215 patients, that patients who received more daily rTMS sessions showed a larger decline in depressive symptoms, with a peak decline at 36 sessions. The findings of these two studies suggest that increasing the number of sessions is more important than increasing amount of pulses to achieve a larger decline in depressive symptoms.

Our study aims to provide a conclusive advice for clinicians using unilateral rTMS protocols for the treatment of TRD. We therefore conducted a meta‐analysis and meta‐regression using the amount of pulses administered and the total number of sessions from RCT's investigating rTMS as a treatment for patients with TRD. Based on the study of Fitzgerald et al. (2020)^10^ and Hutton et al. (2023),^11^ we hypothesized that the total amount of pulses does not show a significant correlation with treatment effect, but that the total number of sessions does. In addition, we investigated other protocol parameters not prescribed in the FDA label, but nevertheless crucial for clinical practice; concomitant medication use, treatment duration, treatment intensity and TMS device.

## 2 METHODS

This meta‐analysis and meta‐regression was executed and written according to PRISMA guidelines.^12^ Our preregistered protocol is available via PROSPERO (CRD42022357668).

### 2.1 Search strategy

We systematically searched the EMBASE, APA PsycInfo, PubMed, and Web of Science databases from inception up until the 20th of September 2024. See DataS2 for complete search strategy. There was no limit on publication date or language to include as many papers as possible. In addition, reference lists of meta‐analyses were checked for potentially relevant articles. After removing duplicates, two independent authors (E. O. and P. J.) performed the title and abstract screening for study eligibility using Rayyan (https://www.rayyan.ai). Full‐text screening and data extraction were performed by E. O. and independently checked by P. J. thereafter.

### 2.2 In‐/exclusion criteria and study selection

We included published and peer‐reviewed RCTs enrolling patients with a primary diagnosis of a depressive episode (within MDD or bipolar disorder) as diagnosed by a clinician or (confirmed) using a structured interview. Trials used unilateral rTMS treatment and were controlled by either a sham condition or another active control condition. The studied rTMS treatment consisted of at least five sessions, with or without add‐on/adjuvant medication. Our primary outcome measure was the difference between baseline and post‐treatment depression severity scores in relation to rTMS protocol parameters (frequency, amount of pulses, number of sessions, inter‐session‐interval, stimulation location). Included protocols consisted of HF‐rTMS stimulating the l‐DLPFC, LF‐rTMS stimulating the r‐DLPFC, or deep‐rTMS (dTMS) stimulating either left (HF) or right (LF) DLPFC. Moreover, protocols deviating from one‐session‐per‐day design were included as well (e.g. more sessions per day or three times per week, etc.). Exclusion criteria were conference abstracts, non‐randomized designs, depression not as a primary diagnosis or another primary neurological diagnosis (e.g. Alzheimer's disease), studies with less than 10 participants included, studies using only bilateral rTMS treatment, intermittent or continuous theta burst stimulation (iTBS and cTBS), only any other form of non‐repetitive TMS or only other neuromodulation techniques (e.g. deep brain stimulation).

Full‐text articles were screened and data extraction was performed using a structured Excel sheet. The following data was extracted from all included studies, if available: (1) treatment effect: baseline and post‐treatment depression severity score, (2) demographics of population: TRD level, (3) study characteristics: inclusion of bipolar patients, allowance of treatment as usual during study treatment duration, (4) rTMS protocol parameters: frequency, amount of pulses, number of sessions, stimulation intensity (%MT), and TMS device. If means or standard deviations (SD) of depression rating scores were not available from the text, they were calculated with the available data or requested by the corresponding author. If the primary outcome was not calculable, unable to extract from another recent meta‐analysis^9^ or when we received no response from the authors, the study was excluded.

### 2.3 Statistical analysis

#### 2.3.1 Primary outcome

Our primary outcome measures were the standardized mean difference (SMD) calculated from baseline and post‐treatment depression severity scores, and the total amount of rTMS pulses and sessions used in the study. If applicable, the sham groups were analyzed separately. Studies were divided in HF protocols and LF protocols. SMD was calculated using formula (4.26) from Borenstein et al.^13^ We used *SD*_*pre_pooled*_ to standardize the differences of mean. This consists of the standard deviation (SD) of the baseline mean depression rating scores from all the study arms used in the study. The *SD*_*pre_pooled*_ was calculated using formula (4.4).^13^ The variance of the SMD is calculated following formula (4.28),^13^ see Data S2 for all formulas. In this analysis, we assume that the correlation coefficient *r* = 0.5^9^, ^14^ for all studies. As a sensitivity analysis, we checked the impact on the effect size when assuming *r* = 0.2 (more conservative), or *r* = 0.8 (less conservative). The calculated standard error of the SMD is the square root of the variance.

#### 2.3.2 Assessment of bias

Individual risk of bias assessment was done for each article by two independent authors (E.O. and P.J.) using the revised ‘Risk of Bias’ tool developed by the Cochrane Collaboration.^15^, ^16^ Any discrepancies were resolved through discussion or by a third independent author (E. v. E.). The studies were examined on the following criteria: random sequence generation; allocation concealment; blinding of participants, study personnel and outcome assessors; missing outcome data; measurement of outcome data; selection of the reported result. In addition, we produced a publication bias assessment by inspecting a funnel plot and conducting Egger's test.

#### 2.3.3 Meta‐regression

Meta‐analyses and meta‐regressions were conducted using the R studio package *metafor*.^17^, ^18^ For our primary analysis, meta‐regressions were performed using a random‐effects model to estimate the effect of the total amount of pulses and total number of sessions (independent variables) on the SMD (dependent variable) between baseline and directly post‐treatment. Instead of correcting the active rTMS outcome with the sham rTMS outcome, we preferred to visualize the relation between pulses and sessions on the one side and treatment outcome on the other side for both active and sham rTMS conditions. Meta‐regression models were created with both the total number of sessions and the total amount of pulses as independent variables to disentangle their effects. We did the same for the total number of sessions and the amount of pulses per session. This was done for both HF‐rTMS studies and LF‐rTMS studies. We used marginal plots for the visualization of the meta‐regression analyses.

#### 2.3.4 Meta‐analyses

Meta‐analyses were performed on the active treatment arms using pre‐post SMD of HF and LF protocols. In case a linear relationship between SMD and the independent variable was violated, we performed subgroup effect size analyses. For this we divided the dataset into quartiles based on the independent variable. In addition, we performed a meta‐analysis using only the RCTs with sham‐rTMS as a control group, using the between group differences (SMD*b*), see Data S2 for the formula.

#### 2.3.5 Secondary and sensitivity analyses

The secondary analyses included the effect of stimulation intensity (%MT), concomitant medication use, total treatment time in workdays, and TMS device. We used a meta‐regression model for the stimulation intensity with SMD as dependent variable and %MT as independent variable for both HF‐rTMS and LF‐rTMS studies. A subgroup analysis within the meta‐analysis was conducted based on concomitant medication use (yes/no), as well as for TMS devices. The effect of treatment duration (total number of workdays) was investigated since we also included studies deviating from the once‐daily treatment design. For this, a meta‐regression model correcting for the total amount of pulses and the total number of workdays was used. The meta‐regressions and meta‐analyses were checked for outliers.

## 3 RESULTS

### 3.1 Search results and characteristics of the included studies

**FIGURE 1:**
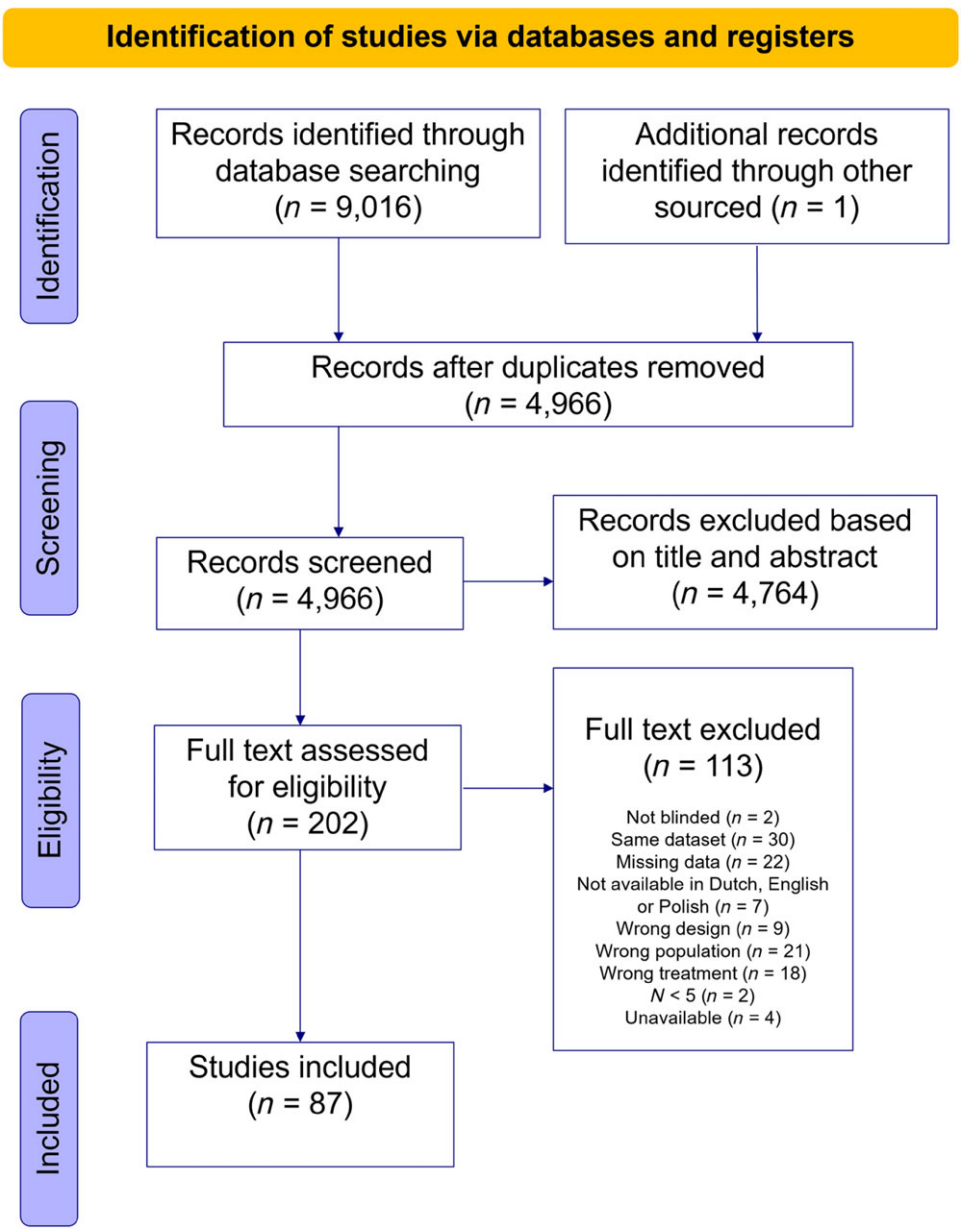
Flowchart of our systematic literature search.

Of the 9016 studies we found with our search, 4966 studies were screened after removing duplicates based on abstract and title, resulting in 202 papers for the full‐text screening (for flow chart, see Figure 1). After full text screening, we included 87 unique RCT studies.^10^, ^19^, ^20^, ^21^, ^22^, ^23^, ^24^, ^25^, ^26^, ^27^, ^28^, ^29^, ^30^, ^31^, ^32^, ^33^, ^34^, ^35^, ^36^, ^37^, ^38^, ^39^, ^40^, ^41^, ^42^, ^43^, ^44^, ^45^, ^46^, ^47^, ^48^, ^49^, ^50^, ^51^, ^52^, ^53^, ^54^, ^55^, ^56^, ^57^, ^58^, ^59^, ^60^, ^61^, ^62^, ^63^, ^64^, ^65^, ^66^, ^67^, ^68^, ^69^, ^70^, ^71^, ^72^, ^73^, ^74^, ^75^, ^76^, ^77^, ^78^, ^79^, ^80^, ^81^, ^82^, ^83^, ^84^, ^85^, ^86^, ^87^, ^88^, ^89^, ^90^, ^91^, ^92^, ^93^, ^94^, ^95^, ^96^, ^97^, ^98^, ^99^, ^100^, ^101^, ^102^, ^103^, ^104^ Sixty‐seven RCTs investigated HF‐rTMS treatment (24 sham‐controlled), eleven RCTs investigated LF‐rTMS treatment (four sham‐controlled), and nine RCTs investigated both HF and LF‐rTMS treatment (four of them also included a sham‐rTMS arm). Every study reported the pre‐ and post‐treatment depression severity score, amount of rTMS pulses, and a number of sessions. If we could not confirm treatment resistance of the study sample, we excluded the RCT. 2478 patients received active HF‐rTMS, 1117 patients received sham HF‐rTMS, 741 patients received active LF‐rTMS, and 139 patients received sham LF‐rTMS (see Table 1).

**TABLE 1:** Demographics and rTMS characteristics of included studies.

HF studies												
	Demographics					rTMS characteristics						
Authors	N exp	N sham	TRD	Bipolar	Med use	Other control	Hz	%MT	#pulses	#sessions	workdays	TMS device
Abdel Latif et al., 2020^19^	20	NA	2	No	Yes	ECT	10	120	50,000	25	25	Magstim
Anderson et al., 2007^21^	13	16	2	No	Yes	NA	10	110	14,000	14	23	Magstim
Armas‐Castañeda et al., 2021 (a)^22^	25	22	NA	No	No	NA	5	110	22,500	15	15	MagPro
Armas‐Castañeda et al., 2021 (b)^22^	22	22*	NA	No	No	NA	5	110	22,500	15	37	MagPro
Asgharian Asl & Vaghef, 2022^23^	14	14	2	No	NA	NA	20	85	25,000	10	10	Magstim
Avery et al., 2006^24^	35	33	1	No	Yes	NA	10	110	24,000	15	20	MagPro
Baeken et al., 2013^25^	9	11	3	No	No	Cross‐over	20	110	31,200	20	4	Magstim
Bakim et al., 2012 (a)^26^	12	12	2	No	Yes	NA	20	80	24,000	30	30	Magstim
Bakim et al., 2012 (b)^26^	11	12*	2	No	Yes	NA	20	110	24,000	30	30	Magstim
Berman et al., 2000^28^	10	10	1	Yes	Yes	NA	20	80	8000	10	10	Cadwell
Blumberger et al., 2012^30^	22	20	2	No	Yes	Bilateral	10	100/120	21,750	15	15	MagPro
Blumberger et al., 2016^29^	40	41	2	No	Yes	Bilateral	10	120	63,000	30	30	MagPro
Blumberger et al., 2018^31^	192	NA	1	No	Yes	iTBS	10	120	60,000	20	20	MagPro
Boutros et al., 2002^32^	12	9	2		Yes	NA	20	80	8000	10	10	Magstim
Bretlau et al., 2008^33^	22	23	2	Yes	Yes	NA	8	90	19,200	15	15	Magstim
Bulteau et al., 2022^35^	60	NA	2	No	Yes	iTBS	10	110	32,000	20	20	MagPro
Chen et al., 2013^37^	10	10	2	no	Yes	NA	20	90	8000	10	10	Magstim
Chen et al., 2021^36^	84	NA	2	Yes	Yes	cTBS/iTBS	10	120	60,000	20	20	Multi
Conca et al., 2002^39^	12	NA	4	Yes	Yes	BL rTMS	10	110	6500	5	5	Cadwell
Dai et al., 2020^40^	62	62	NA	No	Yes	NA	10	100	16,000	20	20	Magstim
Dalhuisen et al., 2024^104^	48	NA	2	No	Yes	Medication	10	120	75,000	25	40	Multi
Eranti et al., 2007^42^	23	NA		Yes		ECT	10	110	15,000	15	15	Magstim
Filipcic et al., 2019^43^	65	NA	2	No	Yes	TAU	10	120	60,000	20	20	Magstim
Filipcic et al., 2021^44^	12	NA	2	No	Yes	NA	18	120	59,400	30	15	Magstim
Fitzgerald et al., 2009 (a)^48^	24	NA	2	No	Yes	5 cm	10	100	30,000	15	15	MagPro
Fitzgerald et al., 2009 (b)^48^	27	NA	2	No	Yes	NN	10	100	30,000	15	15	MagPro
Fitzgerald et al., 2012^50^	24	17	2	No	Yes	BL rTMS	10	120	22,500	15	15	MagPro
Fitzgerald et al., 2018 (a)^49^	60	NA	2	Yes	Yes	NA	10	120	63,000	18	6	NA
Fitzgerald et al., 2018 (b)^49^	59	NA	2	Yes	Yes	NA	10	120	63,000	20	20	NA
Fitzgerald et al., 2020a^46^	38	NA	2	Yes	Yes	TBS	10	120	63,000	20	20	MagPro
Gajsak et al., 2023^53^	51	NA		No	Yes	TAU	18	120	39,600	20	20	Magstim
Garcia‐Toro et al., 2001^54^	17	18	2	No	Yes	NA	20	90	12,000	10	10	MagPro
George et al., 1997^57^	7	5		Yes	Yes	NA	20	80	8000	10	10	Cadwell
George et al., 2010^55^	92	98	3	No	No	NA	10	120	45,000	15	15	Neuronetics
George et al., 2000 (a)^56^	10	10		Yes	No	NA	20	100	16,000	10	10	Cadwell
George et al., 2000 (b)^56^	10	10*		Yes	No	NA	5	100	16,000	10	10	Cadwell
Grunhaus et al., 2002^58^	20	NA	1	No	No	ECT	10	90	24,000	20	20	Magstim
Hernandez‐Ribas et al., 2013^59^	10	11		Yes	Yes	NA	15	100	22,500	15	15	Magstim
Holtzheimer et al., 2004^60^	7	8	2	No	No	NA	10	110	16,000	10	10	MagPro
Jagawat et al., 2022^61^	10	10	2	No	Yes	NA	10	100	30,000	10	10	NA
Jahangard et al., 2019^62^	10	NA		No	Yes	IPL stim	20	100	15,000	10	10	Magstim
Johansson et al., 2021 (a)^64^	12	NA		No	No	NA	18	120	19,800	20	20	Magstim
Johansson et al., 2021 (b)^64^	11	NA		No	No	NA	18	120	39,600	20	20	Magstim
Johansson et al., 2021 (c)^64^	10	NA		no	No	NA	18	120	79,200	20	20	Magstim
Kito et al., 2019 (a)^66^	15	NA	2	Yes	Yes	NA	10	120	84,000	20	20	MagPro
Kito et al., 2019 (b)^66^	15	NA	2	Yes	Yes	NA	10	120	84,000	20	20	MagPro
Li et al., 2020^68^	35	35	1	No	No	piTBS	10	100	16,000	10	10	Magstim
Li, C. et al., 2023^69^	24	24	1	No	No	piTBS	10	120	60,000	20	10	Magstim
Loo et al., 2007^70^	19	19	2	Yes	Yes	NA	10	110	30,000	20	10	Magstim
Manes et al., 2001^72^	10	10	1	No	No	NA	20	80	4000	5	5	Magstim
Martinot et al., 2010^81^	19	14	2	Yes	Yes	PET guided	10	90	16,000	10	10	Magstim
Matsuda et al., 2020^73^	20	20	2	Yes	Yes	NA	18	120	39,600	20	20	Brainsway
McLoughlin et al., 2007^74^	24	NA		Yes	Yes	ECT	10	110	15,000	15	15	Magstim
Miniussi et al., 2005^75^	17	12	2	Yes	Yes	LF left	17	110	2040	5	5	Magstim
Mosimann et al., 2004^76^	15	9	2	Yes	Yes	NA	20	100	16,000	10	10	Magstim
Nahas et al., 2003^77^	11	12		Yes	Yes	NA	5	110	16,000	10	10	Neotonus
O'Reardon et al., 2007^78^	155	146	1	No	No	NA	10	120	60,000	20	20	Neuronetics
Padberg et al., 2002 (a)^79^	10	10	2	NA	Yes	100% MT	10	90	15,000	10	10	Magstim
Padberg et al., 2002 (b)^79^	10	10	2	NA	Yes	90% MT	10	100	15,000	10	10	Magstim
Price et al., 2010^82^	23	NA	1	NA	Yes	rTMS + EEG	10	100	40,000	20	20	Magstim
Ray et al., 2011^83^	31	20		Yes	Yes	NA	10	90	12,000	10	10	Magstim
Rosa et al., 2006^84^	20	NA	2	NA	No	ECT	10	100	50,000	20	20	MagPro
Rossini et al., 2005 (a)^86^	18	17	2	Yes	Yes	80% MT	15	100	6000	10	10	MagPro
Rossini et al., 2005 (b)^86^	19	17	2	Yes	Yes	100% MT	15	80	6000	10	10	MagPro
Su et al., 2005 (a)^89^	10	10	2	Yes	Yes	5 Hz	20	100	16,000	10	10	Magstim
Su et al., 2005 (b)^89^	10	10	2	Yes	Yes	20 Hz	5	100	16,000	10	10	Magstim
Tavares et al., 2017^90^	25	25	2	Yes	Yes	NA	18	120	39,600	20	20	Brainsway
Theleritis et al., 2017 (a)^91^	27	20	2	No	Yes	aTMS	20	100	24,000	15	15	Magstim
Theleritis et al., 2017 (b)^91^	27	24	2	No	Yes	rTMS	20	100	48,000	30	15	Magstim
Tong et al., 2021^92^	60	60	NA	NA	Yes	NA	10	120	40,000	20	20	Magstim
Triggs et al., 2010^93^	18	7	2	No	Yes	Right HF	5	100	20,000	10	10	Magstim
Turnier‐Shea et al., 2006 (a)^95^	8	NA	2	Yes	Yes	weekly	20	100	12,000	10	10	Magstim
Turnier‐Shea et al., 2006 (b)^95^	8	NA	2	Yes	Yes	daily	20	100	6000	5	10	Magstim
Ullrich et al., 2012^96^	22	NA	NA	No	Yes	Left LF	30	110	27,000	15	15	MagPro
van Eijndhoven et al., 2020^97^	15	16	2	No	Yes	NA	10	110	60,000	20	20	Magstim
Wang et al., 2022^99^	29	28	NA	No	Yes	HC	10	120	24,000	10	10	MagPro
Yildiz et al., 2023^100^	15	15	2	No	Yes	HC	10	110	20,000	20	10	Neuronetics
Zengin et al., 2022^101^	14	15	2	Yes	Yes	NA	10	110	20,000	20	10	Neuronetics
Zhang et al., 2021 (a)^102^	55	NA	NA	No	No	BL rTMS	10	100	36,000	30	30	Magstim
Zhang et al., 2021 (b)^102^	53	NA	NA	No	No	BL rTMS	5	100	18,000	30	30	Magstim
Zheng, Li et al., 2010^103^	19	15	2	No	Yes	NA	15	110	60,000	20	20	MagPro
Total (including studies HF + LF arms)	2551	1117		34	66							

HF + LF studies												
	Demographics					rTMS characteristics						
Author	N exp	N sham	TRD	Bipolar	Med use	Other control	Hz	%MT	#pulses	#sessions	workdays	TMS device
Chistyakov et al., 2005 (a)^38^	10	NA	NA	No	No	sham + med/LF	10	100	5000	10	10	Magstim
Chistyakov et al., 2005 (b)^38^	12	NA	NA	No	No	sham + med/HF	3	110	4500	10	10	Magstim
Eche et al., 2012 (a)^41^	8	NA	1	No	Yes	HF	1		2400	20	20	MagPro
Eche et al., 2012 (b)^41^	6	NA	1	No	Yes	LF	10		40,000	20	20	MagPro
Fitzgerald et al., 2003 (a)^45^	20	20	NA	No	Yes	HF	1	100	3000	10	10	Magstim
Fitzgerald et al., 2003 (b)^45^	20	20	NA	No	Yes	LF	10	100	10,000	10	10	Magstim
Fitzgerald et al., 2007 (a)^52^	11	NA	2	No	Yes	HF	1	110	10,800	15	15	MagPro
Fitzgerald et al., 2007 (b)^52^	15	NA	2	No	Yes	LF	10	100	22,500	15	15	MagPro
Fitzgerald et al., 2020b (a)^10^	59	NA	2	Yes	Yes	HF + LF	10	120	45,000	20	20	MagPro
Fitzgerald et al., 2020b (b)^10^	91	NA	2	Yes	Yes	HF + LF	10	120	112,500	20	20	MagPro
Fitzgerald et al., 2020b (c)^10^	59	NA	2	Yes	Yes	HF + LF	1	120	24,000	20	20	MagPro
Fitzgerald et al., 2020b (d)^10^	93	NA	2	Yes	Yes	HF + LF	1	120	72,000	20	20	MagPro
Padberg et al., 1999 (a)^80^	6	6	2	NA	Yes	HF	0,3	90	1250	5	5	Magstim
Padberg et al.,1999 (b)^80^	6	6	2	NA	Yes	LF	10	90	1250	5	5	Magstim
Rossini et al., 2010 (a)^85^	32	NA	1	Yes	Yes	LF	15	100	6000	10	10	Magstim
Rossini et al., 2010 (b)^85^	42	NA	1	Yes	Yes	HF	1	100	6000	10	10	Magstim
Speer et al., 2014 (a)^87^	8	8	2	Yes	No	LF	20	110	24,000	15	15	Cadwell
Speer et al., 2014 (b)^87^	8	8	2	Yes	No	HF	1	110	24,000	15	15	Cadwell
Stern et al., 2007 (a)^88^	10	15	1	No	No	LF	10	110	16,000	10	10	Multi
Stern et al., 2007 (b)^88^	10	15	1	No	No	HF	1	110	16,000	10	10	Multi

LF studies												
	Demographics					rTMS characteristics						
Author	N exp	N sham	TRD	Bipolar	Med use	Other control	Hz	%MT	#pulses	#sessions	workdays	TMS device
Aguirre et al., 2011^20^	19	15	1	No	Yes	NA	1	110	24,000	20	20	MagPro
Bares et al., 2009^27^	27	NA	1	No	No	sham + med	1	100	12,000	20	20	Magstim
Brunelin et al.,2014 (a)^34^	60	NA	NA	No	No	rTMS + med	1	120	10,800	30	30	Multi
Brunelin et al.,2014 (b)^34^	55	NA	NA	No	Yes	rTMS	1	120	10,800	30	30	Multi
Fitzgerald et al., 2006 (a)^51^	67	NA	2	Yes	Yes	2 Hz	1	110	9000	10	10	MagPro
Fitzgerald et al., 2006 (b)^51^	63	NA	2	Yes	Yes	1 Hz	2	110	18,000	10	10	MagPro
Fitzgerald et al., 2011^47^	71	NA	2	Yes	Yes	BL rTMS	1	110	18,000	20	20	MagPro
Januel et al., 2006^63^	11	16			No	NA	1	90	1920	16	20	Magstim
Kaufmann et al., 2004^65^	7	5	2	No	Yes	NA	1	110	1200	10	10	MagPro
Klein et al., 1999^67^	36	34	1	Yes	Yes	NA	1	120	1200	10	10	Cadwell
Mak et al., 2021^71^	26	28	2	Yes	Yes	NA	1	110	4500	15	15	Magstim
Trojak et al., 2014 (a)^94^	8	NA	2	No	No	NA	1	120	3600	10	10	MagPro
Trojak et al., 2014 (b)^94^	7	NA	2	No	No	NA	1	120	3600	10	10	MagPro
Vidya et al., 2023^98^	15	NA	2	No	Yes	prTMS	1	100	12,000	10	10	NA
Total (including studies with HF & LF arms)	741	139		9	16							

%MT, percentage of motor threshold; #pulses, total amount of pulses; #sessions, total amount of rTMS sessions; aTMS, accelerated TMS; BL, bilateral; cTBS, continuous theta burst stimulation; ECT, electroconvulsive therapy; EEG, electroencephalography; exp, experimental arm; HF, high frequency; Hz, Herz; iTBS, intermittent theta burst stimulation; IPL, inferior parietal lobe; LF, low frequency; Med, medication; NA, not available; NN, neuronavigation; PET, positron emission tomography; rTMS, repetitive transcranial magnetic stimulation; stim, stimulation; TAU, treatment as usual; TRD, treatment resistant depression.

* same sham group as mentioned in the other arm of the same study.

### 3.2 Results of the meta‐regressions

#### 3.2.1 Models with sessions and total amount of pulses as independent variables

**FIGURE 2:**
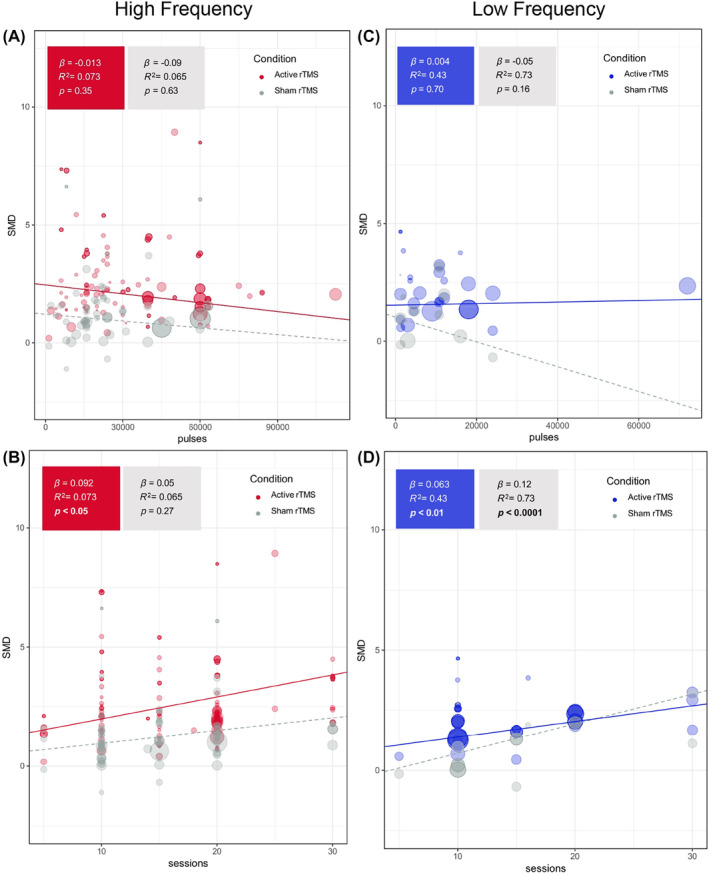
Meta‐regressions of the total amount of pulses or total amount of sessions as independent variable and SMD as dependent variable. (A): Meta‐regression of HF‐rTMS studies between total amount of pulses and SMD, corrected for total number of sessions. (B): Meta‐regression of HF‐rTMS studies between total number of sessions and SMD, corrected for total amount of pulses. (C): Meta‐regression of LF‐rTMS studies between total amount of pulses and SMD, corrected for total number of sessions. (D): Meta‐regression of LF‐rTMS studies between total number of sessions and SMD, corrected for total amount of pulses. HF, high‐frequency; LF, low‐frequency; rTMS, repetitive transcranial magnetic stimulation; SMD, standardized mean difference.

Both the total number of sessions and the total amount of pulses were used as independent variables in one model. We found a statistically significant positive correlation between SMD and total number of sessions for active HF‐rTMS (*β* = 0.09, tau = 1.39, *p* < 0.05, *R* ^2^ = 0.073); and for both active (*β* = 0.06, tau = 0.54, *p* < 0.01, *R* ^2^ = 0.45) and sham LF‐rTMS (*β* = 0.12, tau = 0.57, *p* < 0.0001, *R* ^2^ = 0.72). Total amount of pulses did not show a statistically significant correlation with SMD for both HF‐ and LF‐rTMS studies (see Figure 2).

#### 3.2.2 Models with sessions and pulses per session as independent variables

Here the total number of sessions and the amount of pulses per session were used as independent variables in one model. Statistical significance was reached for the correlation between the SMD and total number of sessions for the active HF‐rTMS (*β* = 0.08, tau = 1.38, *p* < 0.05, *R* ^2^ = 0.073) and for both active (*β* = 0.07, tau = 0.54, *p* < 0.01, *R* ^2^ = 0.43) and sham rTMS (*β* = 0.10, tau = 0.60, *p* < 0.001, *R* ^2^ = 0.73) of the LF studies. No statistically significant correlation was found for pulses per session for both HF and LF models, as shown in Figure S1.

### 3.3 Meta‐analyses

**FIGURE 3:**
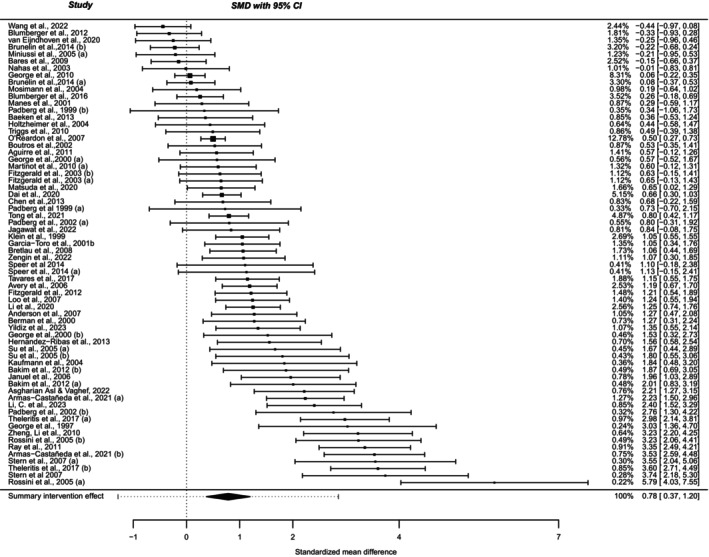
Forest plot of the randomized clinical trials (RCTs) using a sham control arm. Using the SMD calculated between groups enables us to interpret these results corrected for sham‐effect. An effect size of 0.78 was found, which favors active treatment over sham rTMS treatment. CI, confidence interval; rTMS, repetitive transcranial magnetic stimulation; SMD, standardized mean difference.

Since no linear relation was found between the total amount of pulses and the number of pulses per session and the SMD for both HF‐rTMS and LF‐rTMS, we divided the dataset into quartiles based on the total amount of pulses and the number of pulses per session and performed subgroup effect size analyses between these quartiles. Furthermore, we conducted a meta‐analysis using only sham‐controlled RCTs, resulting in an effect size of 0.78 (0.37–1.20, *p* < 0.0001), see Figure 3.

#### 3.3.1 Subgroup effect size analyses based on total pulses

The HF‐rTMS studies showed the largest subgroup effect size for studies applying between 15,000 and 22,500 pulses in total, which was 2.46 (SE = 0.22; *p* < 0.0001). However, all the subgroup effect sizes were statistically significant and overlapped (Figure S2). The LF‐rTMS studies showed the highest subgroup effect size for studies applying between 10,800 and 12,000 pulses in total, which was 2.40 (SE = 0.58; *p* < 0.0001). The other subgroup effect size analyses were also statistically significant, except for the studies applying ≤3000 pulses. See Table 2A and Figure S3 for more details.

**TABLE 2:** Sub‐summary intervention effects for total amount of pulses and pulses per session.

A: Total amount of pulses							
	Effect (SE)	CI	τ	*I* ^2^	*H* ^2^	*z*	*p*
HF							
≤15,000	1.82 (0.64)	0.57–3.07	1.86	90.26%	10.27	2.86	**0.0043**
>15,000 ≤ 22,500	2.46 (0.22)	2.04–2.89	0.73	57.45%	2.35	11.42	**< 0.0001**
>22,500 ≤ 40,000	2.26 (0.37)	1.55–2.97	1.01	79.83%	4.96	6.23	**< 0.0001**
>40,000	2.10 (0.64)	0.84–3.36	1.96	97.22%	35.95	3.26	**0.0011**
LF							
≤3000	1.33 (0.71)	−0.06–2.72	1.28	89.91%	9.91	1.87	0.062
>3000 ≤ 9000	1.55 (0.23)	1.11–1.99	0.33	50.71%	2.03	6.86	**<0.0001**
>9000 ≤ 12,000	2.40 (0.30)	1.82–2.99	0.60	72.46%	3.63	8.05	**<0.0001**
>12,000	1.88 (0.41)	1.07–2.69	0.84	91.60%	11.90	4.56	**<0.0001**

B: Pulses per session							
	Effect (SE)	CI	*τ*	*I* ^2^	*H* ^2^	*z*	*p*
HF							
≤ 1000	1.97 (0.58)	0.83–3.11	1.77	90.26%	10.26	3.38	**0.0007**
≥1200 < 1600	2.53 (0.32)	1.90–3.15	1.08	70.40%	3.38	7.92	**< 0.0001**
≥1600 < 2000	2.12 (0.33)	1.47–2.76	0.95	79.42%	4.86	6.41	**< 0.0001**
≥2000	2.14 (0.57)	1.02–3.26	1.81	96.21%	26.39	3.73	**0.0002**
LF							
≤300	1.39 (0.52)	0.38–2.41	1.04	87.48%	7.99	2.70	**0.0069**
>300 < 500	2.45 (0.38)	1.71–3.19	0.73	75.41%	4.07	6.49	**< 0.0001**
≥500 < 1000	1.76 (0.25)	1.26–2.25	0.42	72.03%	3.58	6.96	**< 0.0001**
≥1000	1.80 (0.46)	0.89–2.70	0.86	90.41%	10.43	3.90	**< 0.0001**

*Note*: Datasets were divided based on quartiles of the total amount of pulses (A) and pulses per session (B). Statistical significance was reached when *p* < 0.05.

Abbreviations: CI, 95% confidence interval; HF, high frequency; LF, low frequency; SE, standard error.

#### 3.3.2 Subgroup effect size analyses based on pulses per session

The HF‐rTMS studies that showed the highest subgroup effect size (2.53, SE = 0.32, *p* < 0.0001) applied between 1200 and 1500 pulses per session. However, the other subgroup effect sizes also showed statistical significance and overlapped (Figure S4). The LF‐rTMS studies that applied more than 300 but less than 500 pulses per session showed the highest subgroup effect size (2.45, SE = 0.74, p < 0.0001). The other subgroup effect sizes also showed statistical significance, although all had SMDs below 2. See Table 2B and Figure S5 for more details.

### 3.4 Secondary analyses

#### 3.4.1 Stimulation intensity

**FIGURE 4:**
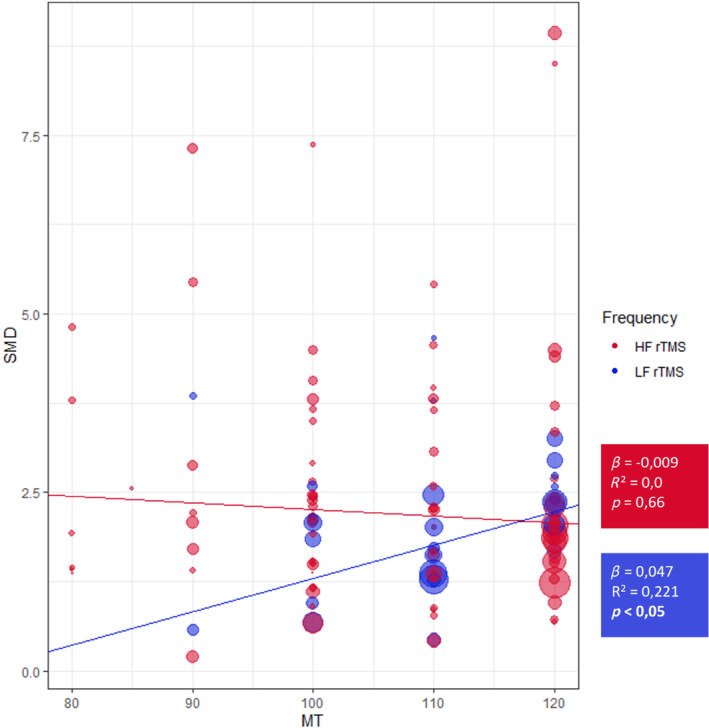
Meta‐regressions between stimulation intensity and SMD, for both HF‐rTMS and LF‐rTMS. Regression analysis between the % MT and the SMD of the active HF‐rTMS (in red) and active LF‐rTMS (in blue). Statistical significance was reached when *p* < 0.05. HF, high‐frequency; LF, low‐frequency; rTMS, repetitive transcranial magnetic stimulation; SMD, standardized mean difference.

Using %MT as an independent variable of interest in a meta‐regression model for both HF‐rTMS and LF‐rTMS resulted in a statistically significant positive correlation between SMD and %MT for LF‐rTMS (*β* = 0.05, *p* < 0.05, *R* ^2^ = 0.22). Still, no significance was reached for HF‐rTMS, as shown in Figure 4 and Table 3B.

**TABLE 3:** Secondary analyses on medication use, stimulation intensity and treatment duration in workdays.

A: Subgroup effect size analyses based on medication use							
	Effect (SE)	CI	*τ*	*I* ^2^	*H* ^2^	*z*	*p*
HF							
No medication	2.34 (0.34)	1.68–3.00	1.37	86.88%	7.62	6.91	**< 0.0001**
With medication	2.52 (0.19)	2.14–2.91	1.47	91.35%	11.57	12.98	**< 0.0001**
LF							
No medication	2.33 (0.44)	1.47 3.18	1.08	83.84%	6.19	5.32	**< 0.0001**
With medication	1.84 (0.17)	1.50 2.18	0.60	82.27%	5.64	10.62	**< 0.0001**

B: Meta‐regression with stimulation intensity as independent variable							
	*β* (SE)	*τ*	*I* ^2^	*H* ^2^	*R* ^2^	*z*	*p*
HF							
Active	−0.0093 (0.021)	1.45	90.66%	10.71	0.000	−0.44	0.6592
LF							
Active	0.047 (0.022)	0.68	82.96%	5.87	0.221	2.08	0.0276*

C: Meta‐regression with total treatment time and pulses as independent variables							
HF	*β* (SE)	*τ*	*I* ^2^	*H* ^2^	*R* ^2^	*z*	*p*
Workdays							
Active	0.055 (0.04)	1.42	90.00%	10.00	0.26	1.40	0.1616
Sham	0.031 (0.03)	0.95	91.19%	11.36	0.042	1.06	0.288

D: Meta‐regression with total treatment time and pulses/session as independent variables							
	*β* (SE)	*τ*	*I* ^2^	*H* ^2^	*R* ^2^	*z*	*p*
Workdays							
HF							
Active	0.051 (0.033)	1.41	89.85%	9.85	0.040	1.56	0.120
Sham	0.029 (0.025)	0.95	91.15%	11.30	0.042	1.15	0.249

Note: (A) Subgroup effect size analyses were done using allowance of medication use (yes/no). Meta‐regression analyses were done using SMD as dependent variable and either (B) stimulation intensity, (C) treatment time in workdays and pulses or (D) treatment time in workdays and pulses per session as covariates. Statistical significance was reached when *p* < 0.05.

Abbreviations: CI, 95% confidence interval; HF, high frequency; LF, low frequency; SE, standard error.

#### 3.4.2 Concomitant medication use

We found a subgroup effect size of 2.34 (SE = 0.34, *p* < 0.0001) for HF‐rTMS without medication and 2.52 (SE = 0.19, *p* < 0.0001) with concomitant medication. LF‐rTMS studies without concomitant medication use showed a subgroup effect size of 2.33 (SE = 0.44, *p* < 0.0001), and studies that allowed concomitant medication use showed an effect size of 1.84 (SE = 0.17, *p* < 0.0001). These effect sizes all overlapped, see Table 3A for more details.

#### 3.4.3 Total treatment time

Fourteen HF‐rTMS studies deviated from the conventional once‐daily protocol design (five times a week); eight studies used an accelerated protocol,^25^, ^44^, ^49^, ^69^, ^70^, ^91^, ^100^, ^101^ and six studies administered rTMS less than once‐daily.^21^, ^22^, ^24^, ^63^, ^95^, ^104^ All of the LF‐rTMS studies used the conventional once‐daily design, five times a week. Therefore, no additional regression analyses between treatment time and SMD were performed for LF‐rTMS studies. The total number of sessions of the HF‐rTMS studies showed a strong correlation with duration of treatment expressed in number of workdays (*r* = 0.77, t(87) = 11.13, *p* < 0.0001). No statistically significant relationship was found between SMD and total treatment duration in workdays when we corrected for the total amount of pulses, or pulses per session, for either sham or active HF‐rTMS. See Table 3C,D for more details.

#### 3.4.4 TMS devices

Only four studies did not specify which device they used. The majority of the studies used a Magstim device (*n* = 42), a MagPro device was used by 25 studies (combined Magventure, Medtronic and Dantec), six studies used a Cadwell device, five studies used a Neurentics device, Brainsway was used by two studies and six studies used multiple devices (see Table 1). The highest effect size was found for studies using multiple devices however the effect sizes all overlapped (see Table S1).

#### 3.4.5 Publication bias & sensitivity analyses

**FIGURE 5:**
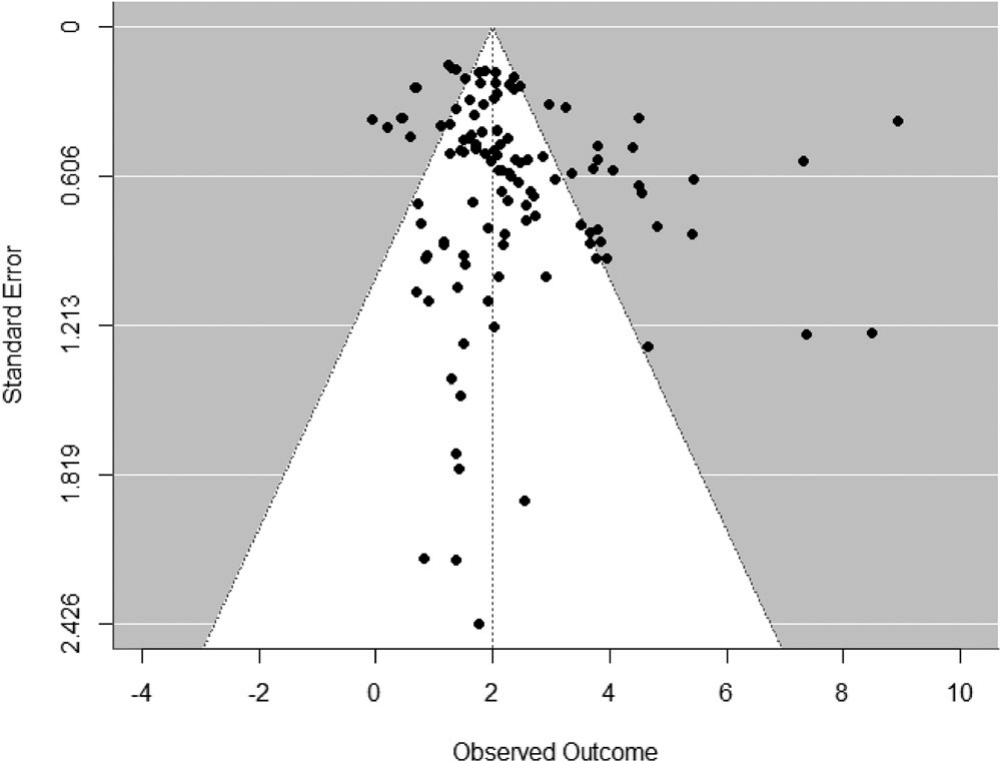
Funnel plot.

A funnel plot was used to determine the possibility of publication bias, see Figure 5. Egger's test showed a significant correlation (b = 1.46, t(114) = 3.13, *p* < 0.01), indicating publication bias. Additionally, we conducted sensitivity analyses without outliers and different correlation coefficients. See Data S3 and S3.2 for the results.

## 4 DISCUSSION

This meta‐regression and meta‐analysis aimed to investigate the optimal rTMS parameters for TRD treatment. We hypothesized that the treatment effect size depends on the number of sessions rather than the amount of pulses. The total number of sessions showed a positive correlation with the SMD. The total amount of pulses and pulses per session did not correlate with the SMD of the active HF‐rTMS and LF‐rTMS studies. This suggests that we should increase the number of sessions rather than the amount of pulses in rTMS treatment for TRD, to increase the chance of treatment efficacy.

### 4.1 No correlation between SMD and amount of pulses

The largest SMD's were found for 1200 to 1500 pulses per HF‐rTMS session and between 15,000 and 22,500 pulses in total. For LF‐rTMS, the largest SMD's were found for 360 to 450 pulses per session and between 10,800 and 12,000 pulses in total. However, these SMD's did not differ significantly when we compared them with a higher or lower range of pulses per session. This indicates that the SMD reaches plateau. Our findings are in line with other studies that did not find improved treatment outcomes when increasing the amount of pulses.^9^, ^10^, ^105^, ^106^, ^107^ In addition, a recent dose–response meta‐analysis was able to show that receiving more HF‐rTMS pulses resulted in a worse outcome, with peak efficacy at 12,374 (95% CI 11,185‐15,026) pulses.^108^ This is lower than our results; however this study exclusively included 26 RCTs that investigated HF‐rTMS. This resulted in a smaller range of pulses administered; they included only four studies that used >40,000 pulses, with 60,000 being the highest amount.

The neurobiological mechanism behind the effects of rTMS is probably caused by rTMS‐induced plasticity.^109^, ^110^, ^111^ This most likely includes modulation of synaptic plasticity and homeostatic plasticity. The latter is the capability of neurons to maintain a stable inhibition and excitation balance and to return, after disturbance, to a state of equilibrium.^112^, ^113^ In silico homeostatic plasticity has recently been shown to be sensitive to the amount of rTMS pulses and exhibits a plateau in rTMS‐induced changes in connectivity.^114^ This could be an explanation why we see a plateau; but, more clinical research into this effect of saturation is necessary to optimize rTMS treatment.

### 4.2 More rTMS sessions result in higher SMD

We found a statistically significant correlation between the number of sessions and SMD for HF‐rTMS protocols and LF‐rTMS protocols. We found a large effect, that is, 0.9 points of difference in SMD, between 20 and 30 HF‐rTMS sessions, and a medium to large effect, that is, 0.6 points of difference in SMD, between 20 and 30 LF‐rTMS sessions. Several clinical studies regarding rTMS treatment in TRD also showed that applying more sessions was followed by more patients responding to rTMS.^11^, ^106^, ^115^, ^116^ A clinical explanation for this could be that patients show different response curves, for example, a steep decline at the start following a slower but steady decline in symptoms (rapid response) or an overall slow but steady decline (linear response).^115^ As a consequence of the different response trajectories, it can be relatively difficult to predict non‐response in the early treatment phase; causing clinicians to prematurely terminate the treatment because of lack of response.^11^, ^117^ Extending the number of treatment sessions can result in higher treatment response rates. Additionally, Berlow et al. 2023^118^ found that the trajectories of rapid responders follow a more exponential decay function, rather than a linear function. Whereas slow responders corresponds more with a linear response. This underlines the differences in amount of sessions needed to respond between patients with rapid response versus linear response.

Another explanation for the significant contribution of rTMS sessions to improved outcome, could be related to rTMS‐induced neuroplasticity. These effects appear to be short‐term after one session, but long‐term after multiple sessions.^109^ This would imply that short‐term changes convert into long‐term changes when more sessions are administered. Such neuroplasticity typically plays out over time and one could a priori expect that the time duration in which the treatment occurs, predicts treatment effect; our results nevertheless indicate that, rather than the number of days, the number of sessions predicts the treatment effect. It would be of interest in future analyses to investigate the interaction between number of sessions and the duration of the treatment in predicting optimal consolidation of the rTMS effect.

### 4.3 Effect of stimulation intensity

In the studies with patients receiving LF‐rTMS, we found that an increased number of sham rTMS sessions also contribute to an increase in the treatment effect. This suggests that the number of sessions contributes similarly to treatment efficacy in both sham and active LF‐rTMS. This effect was only found in patients treated with LF‐rTMS. We present two possible explanations for this result. First, important to keep in mind is the lower number of patients that received sham‐rTMS for LF‐rTMS compared with HF‐rTMS studies (*n* = 113 vs. *n* = 1117, respectively). Second, only at 120% MT, we found a similar treatment effect in LF‐rTMS studies as in the HF‐rTMS studies. This suggests that studies that used <120% MT probably have been understimulating patients, which could result in not more than a placebo effect.

### 4.4 Strengths and limitations

One of the limitations of this meta‐analysis is the high heterogeneity observed between studies. It is known that rTMS treatment has been studied in various ways, with different frequencies, coils, clinical instruments to assess depression severity, number of sessions, number of pulses, treatment intensities, and so on. Therefore, we limited our search to the two most common clinically used unilateral protocols and decided not to include faster protocols such as iTBS, cTBS, or bilateral stimulation. This heterogeneity is not only a negative feature of the rTMS treatment research field; this variety of protocols allows us to compare designs and find the optimal parameters in treating TRD. Our second limitation is the possible publication biased indicated by the asymmetry observed in the funnel plot and significant Egger's test. However, funnel plots require caution with interpretation since tests for publication bias might be unsuitable with substantial heterogeneity (*I* ^2^ >50%).^119^ In addition, Dalhuisen et al. (2022)^9^ suggested with their meta‐regression that the significant tests for publication bias are the results of substantial heterogeneity instead of publication bias. Thirdly, we used RCTs that included sham‐rTMS as a control group and RCTs that included multiple active arms without a sham arm. This could result in more biased outcomes on study level, since the raters knew the participant received an active treatment. However, this approach allowed us to use a larger sample and include all RCTs, which resulted in a larger range of our primary outcome measures. Furthermore, we did not analyze the long‐term efficacy and risks of relapse over time; we only analyzed treatment effect direct post‐treatment. More research on long‐term efficacy of rTMS treatment and chances in relapse is necessary. At last, we did not include the type of heuristic used to target the DLPFC as a variable in the analyses. A few studies found differences in treatment response between the 5 cm rule and neuronavigation.^48^, ^120^ However, even though the 5 cm rule and the Beam F3/F4 method differed significantly in terms of inter‐ and intra‐rater reliability,^121^ no clinical significant differences were found.^122^ A large RCT is necessary to study the possible differences in depression outcome and cost‐effectiveness between neuronavigation methods and 5 cm rule or Beam F3/F4 method.

### 4.5 Implication and Conclusion

Considering our notable results, we advise to use a minimum of 30 sessions in the rTMS treatment for TRD, contrary to the FDA approved protocol. In addition, our study suggests that 3000 pulses per session is more than sufficient; our findings even indicate that the number of pulses per session and the total amount of pulse could be lowered. Therefore, in contrast to the FDA approved protocol, one could administer a minimum of 1200 HF‐rTMS pulses per session, with the clinical benefit of the option to reduce the session duration in time. In addition, it can be considered to reduce stimulation intensity of HF‐rTMS when a patient cannot handle a stimulation of 120% MT, since our data suggest similar efficacy for HF‐rTMS at lower MT. At last, we propose the incorporation of LF‐rTMS protocol stimulating the right DLPFC as treatment of TRD, with a minimum of 30 sessions and at least 360 LF pulses at a stimulation intensity of 120% MT.

With our meta‐regression using 87 unique RCT studies, we aimed to establish optimal rTMS treatment parameters for treating TRD. To our knowledge, this is the first time the number of sessions and amount of pulses for both active and sham rTMS protocols have been analyzed separately. While more research is still needed into the mechanism of rTMS‐induced neuroplasticity to find the optimal amount of rTMS pulses, long‐term efficacy and risks in relapse, our main advice on the basis of the current investigation is to apply more sessions into standard rTMS treatment for TRD.

## AUTHOR CONTRIBUTIONS

**E. Oostra**: Conceptualization, data curation, formal analysis, investigation, methodology, validation, project administration, resources, visualization, writing—original draft, writing—review and editing. **P. Jazdzyk**: Data curation, validation, investigation. **V. Vis**: Methodology, formal analysis, investigation. **I. Dalhuisen**: Resources, writing—review and editing. **A. W. Hoogendoorn**: Methodology, formal analysis, investigation, supervision, writing—review and editing. **C. H. M Planting**: Software, resources, project administration **P.F. van Eijndhoven**: Conceptualization, resources, writing—review and editing. **Y. D. van der Werf**: Conceptualization, supervision, writing—review and editing. **O. A. van den Heuvel**: Conceptualization, writing—review and editing, supervision, project administration. **E. van Exel**: Conceptualization, investigation, validation, methodology, writing—review and editing, supervision.

## CONFLICT OF INTEREST STATEMENT

The authors report no biomedical financial interests or potential conflicts of interests.

### PEER REVIEW

The peer review history for this article is available at https://www.webofscience.com/api/gateway/wos/peer‐review/10.1111/acps.13768.

## Supporting information


**Data S1.** Dataset_pulsesvssessions_Oostraetal.

**Data S2.** Supporting Information.

## Data Availability

The data that supports the findings of this study are available in the supplementary material of this article.
