# Supplementary material for: More rTMS pulses or more sessions? The impact on treatment outcome for treatment resistant depression
Source: Acta Psychiatr Scand. 2024 Nov 21;151(4):485–505. doi: 10.1111/acps.13768 (PMC11884915; doi:10.1111/acps.13768)
Supplement: Supplementary file 1 — Data S1. Dataset_pulsesvssessions_Oostraetal. [file ACPS-151-485-s002.pdf]

Table A: study arms with data of participants receiving HF rTMS protocol

Author	Baseline	SD	Post	SD	Baseline Sham	SD	post Sham	SD
**Abdel Latif et al,, 2020**	40,5	1,36	23,36	6,19				
**Anderson et al,, 2007 ***	26,7	3,6	15	9,7	27,7	7,1	23,4	9,8
Armas-Castañeda et al,, 2021 (a)	26,76	4,08	6,6	4,1480	27,23	4	16,95	6
Armas-Castañeda et al,, 2021 (b)	29,36	5,41	5,41	3,402	27,68	4,43	19,36	4
Asgharian Asl & Vaghef, 2022	36,25	8,324	11,42	5,282	37,42	11	34,08	11
Avery et al,, 2006	23,5	3,9	15,7	7,8	23,5	2,9	19,8	6,3
Baeken et al,, 2013	27,13	8,34	20	8,94	26,45	8,71	22,36	10,01
Bakim et al,, 2012 (a)	23,08	3,63	10,17	7,42	25,58	3,82	19,5	7,83
Bakim et al,, 2012 (b)	24,09	2,77	11,64	8,12				
**Berman et al,, 2000**	37,1	9,7	24,6	9,22	37,3	8,47	36,4	9,05
Blumberger et al,, 2012	26	3,3	19,6	5,6	25,2	2,8	17,8	4,5
Blumberger et al,, 2016	26	3,4	19,6	7	25,5	3,6	20,5	4,8
Blumberger et al,, 2018	23,5	4,4	13,4	7,8				
**Boutros et al,,2002**	34,40	10,10	29,08	14,34	31,70	4,90	28,11	13,92
Bretlau et al,, 2008	25,3	3	16,4	4,5	24,7	3,2	19,1	4,8
Bulteau et al,, 2022 **#**	27,3	3,3	15,52	3,11				
**Chen et al,,2013**	23,5	1,9	9,6	1,5	24,9	1,9	12,3	1,4
Chen et al,,2021	16,58	3,82	9,4	5,58				
**Chistyakov et al,, 2005 (c)**	27,3	5,3	21,39	8,69				
**Conca et al,, 2002**	30,2	5,7	21,4	6,7				
**Dai et al,, 2020**	33,97	4,95	14,35	3,95	33,43	4,95	17,24	5,21
Dalhuisen et al,, 2024	21,6	4,1	11,58	7,78				
**Eche et al,, 2012 (b)**	29,8	6,9	19,38	6,7002				
**Eranti et al,, 2007(b) #**	23,9	7	18,73	1,58				
**Filipcic et al,, 2019 (b)**	17	5,3	6	5,1				
**Filipcic et al,, 2019(a)**	17	5,4	10	6,9				
**Filipcic et al,, 2021 (a)**	22	3,3	9	3,8				
**Filipcic et al,, 2021 (b)**	20	2,4	9	3,8				
**Fitzgerald et al,, 2003 (b)**	36,1	7,5	30,8	7,5	35,7	8,1	35,4	7,5
Fitzgerald et al,, 2007 (b)	34,5	4,9	24,8	9,57				
**Fitzgerald et al,, 2009 (a)**	30,8	4,5	21,5	11,4				
**Fitzgerald et al,, 2009 (b)**	33,4	7,3	26,2	11,2				
**Fitzgerald et al,, 2012**	23,7	3,8	19,6	4,2	22,8	2,1	22,6	5
Fitzgerald et al,, 2018 (a)	31,3	5,3	23,2	9,2				
**Fitzgerald et al,, 2018 (b)**	31,6	5,4	21,6	10,5				
**Fitzgerald et al,, 2020a**	31,9	6,7	27,6	7,9				
**Fitzgerald et al,, 2020b (a)**	26,90	6,0	12	9,8				
**Fitzgerald et al,, 2020b (b)**	26,1	6,7	13	9,73				

**Gajsak et al,, 2023**	17	5,41	7,4	6,29				
**Garcia-Toro et al,, 2001b**	27,11	6,65	18,94	7,69	25,6	4,92	23,55	6,07
George et al,, 1997	30	4	23	9	26	3	30	8
George et al,, 2010	26,26	4,95	21,61	9,26	26,5	4,8	23,38	7,43
George et al,,2000 (a)	30	5,8	22,2	10,6	23,8	4,1	19	6
George et al,,2000 (b)	26,3	5,9	13,5	5,8				
**Grunhaus et al,, 2002**	24,4	3,9	13,3	9,2				
**Hernandez-Ribas et al,, 2013**	19,7	3,8	8,7	5,06	16,55	2,4	10,45	4,7
Holtzheimer et al,, 2004	22,7	5,3	14,6	3,2	20,8	6,3	15,3	3
Jagawat et al,, 2022	15	2,67	9,63	4,31	14,57	2,3	11,29	3,04
Jahangard et al,, 2019	35,6	5,56	27,7	4				
**Johansson et al,, 2021 (a)**	27	4	15,4	10				
**Johansson et al,, 2021 (b)**	27,9	8,2	18,8	12,8				
**Johansson et al,, 2021 (c)**	27,4	4,3	16,1	8,5				
**Kito et al,, 2019 (a)**	13,5	2,6	7,7	4,1				
**Kito et al,, 2019 (b)**	14,2	2,9	8,3	5,6				
**Li et al,, 2020**	22,9	3,8	15,114	4,2	23,1	3,5	19,89	4,2
Li, C, et al,, 2023	25,6	1,3	15,7	1,5	24,1	1,2	17	1,1
Loo et al,, 2007	29,5	3,9	18,9	7,7	32,6	4,3	27,1	10,2
Manes et al,, 2001	22,7	5,2	13,7	5,4	22,7	7,1	15,5	9,1
Martinot et al,, 2010 (a)	32	7,78	17,28	8,94	34,57	6,07	24,07	12,34
Matsuda et al,, 2020	19,4	8,2	14,95	7,4464	20,5	4,1	20,28	7,521
McLoughlin et al,, 2007	23,9	7	18,5	7,1898				
**Miniussi et al,, 2005 (a)**	19,4	4,43	12,61	4				
**Mosimann et al,, 2004**	28,5	4,6	23,3	7,2	24,5	7,3	20,4	6,6
Nahas et al,, 2003	32,5	4,3	24,375	10,4	32,8	7,6	24,6	10
O'Reardon et al,, 2007	22,6	3,3	17,4	6,5	22,9	3,5	19,4	6,5
Padberg et al,, 1999 (b)	30,2	9,5	28,5	9,4	22,2	8,8	23,5	10,4
Padberg et al,, 2002 (a)	21,9	1,8	18,64	1,95	24,4	2,1	22,67	1,42
Padberg et al,, 2002 (b)	23,6	1,9	16,61	2,05				
**Price et al,, 2010**	23,4	6,6	16,8	9				
**Ray et al,, 2011**	29,6	3,79	4,5	5,23	29,4	5,66	19,75	6,43
Rosa et al,, 2006 **#**	30,1	4,7	20,83	5,37				
**Rossini et al,, 2005 (a)**	28,8	3,1	9	6,70022	28,7	2,1	24,515	6,46157
Rossini et al,, 2005 (b)	28,6	2,7	16	6,70022				
**Rossini et al,, 2010 (a)**	24,7	4,89897	11,3	6,70022				
**Speer et al,, 2014 (a)**	35,8	10,6	32,5	10,1	24	4,6	29,3	6
Stern et al,, 2007 (a)	27,8	3,2	15,1	6	27,4	2,9	26,7	3,6
Su et al,, 2005 (a)	23,2	7,5	9,8	7,1	22,7	4,7	19	7,7
Su et al,, 2005 (b)	26,5	5,2	12,3	7,7				
**Tavares et al,, 2017**	25,8	5,25	13,5	9,41	25,32	3,76	18,26	9,88
Theleritis et al,, 2017 (a)	30,6	3,2	15,6	3,7	29,4	3,2	25,4	5,3
Theleritis et al,, 2017 (b)	29,7	4,6	13,1	4,5	30,3	3,6	27	4

Tong et al,, 2021	29,63	3,55	12,68	2,78	28,5	3,97	14,55	2,44
Triggs et al,, 2010	28,2	6	19,8	9,1	27,7	3,5	22	11,6
Turnier-Shea et al,, 2006 (a)	23,3	3,6	13	6				
**Turnier-Shea et al,, 2006 (b)**	22	3,5	14,5	3,8				
**Ullrich et al,, 2012**	30,4	4,8	23,1	5,7				
**van Eijndhoven et al,, 2020**	24,1	4,2	21	5,4	22,7	3,8	18,6	4,2
Wang et al,, 2022	28,5	3,8	14,41	2,97	33,1	4,6	17,14	3
Yildiz et al,, 2023	22,2	4,85	7,13	3,9	20,2	4,96	11,73	4,75
Zengin et al,, 2022	20,4	2,8	14,2	5,7	20,1	2,6	16,8	2,8
Zhang et al,, 2021 (a)	29,9	5,2	16,9	7,3				
**Zhang et al,, 2021 (b)**	30,3	5,7	16,9	6,7				
**Zheng, Li et al,, 2010**	24,6	3	13,5	5,1	24,6	2,8	22,9	3,4

When SD's were not available, calculatable and could not be retrieved from the authors, we used the mean SD for that dataset (for example, HF dataset, pre-treatment SD). These SD's are written in red.

means data retrieved from figures in paper

***** means data retrieved from author when requested

Table B: study arms with data of participants receiving LF rTMS protocol

Author	Baseline	SD	Post	SD	Baseline Sham	SD	post Sham	SD
**Aguirre et al,, 2011 ***	21,3684	3,8280	14,4444	4,3234	20,3333	4,4372	15,7333	4,0078
Bares et al,, 2009	27,5	4,1	20	9,3	26,7	4	18,6	8,6
Brunelin et al,,2014(a)	25,8	3,6	14	7,8725				
**Brunelin et al,,2014(b)**	26,1	3,9	15,4	7,8725				
**Chistyakov et al,, 2005 (b)**	26,8	6,7	20,05	8,86				
**Eche et al,, 2012 (a)**	32	8	24,9	7,8725				
**Fitzgerald et al,, 2003 (a)**	37,7	8,4	32,2	9	35,7	8,1	35,4	7,5
Fitzgerald et al,, 2006b (a)	24,13	4,87	17,75	7,82				
**Fitzgerald et al,, 2006b (b)**	22,62	5,06	15,84	8,9				
**Fitzgerald et al,, 2007 (a)**	33,3	3,8	25,6	9				
**Fitzgerald et al,, 2011**	21,8	4,7	9,02	4,86				
**Fitzgerald et al,, 2020b (c)**	25,8	6,3	13,0	9,8				
**Fitzgerald et al,, 2020b (d)**	25,7	5,8	11	12,3				
**Januel et al,, 2006**	21,73	3,52	9,9	5,95	22,5	2,73	16,69	4,61
Kaufmann et al,, 2004	21,86	2,31	11,29	3,17	18,2	2,2	11,8	1,93
Klein et al,, 1999	25,8	5,6	13,7	9,2	25,3	6,4	19,7	10,3
Mak et al,, 2021	27,5	5,2	19,19	10,3				
**Padberg et al 1999 (a)**	26,7	9,4	21,5	21,5	22,2	8,8	23,5	10,4
Rossini et al 2010 (b)	24,3	5,1960	13,8	7,8725				
**Speer et al 2014**	28,6	7,6	25,1	6,9	24	4,6	29,3	6
Stern et al 2007	27,9	3,8	15,8	4,8	27,4	2,9	26,7	3,6
Trojak et al 2014	26,25	1,67	16,87	8,04				
**Trojak et al 2014**	30,29	4,72	21,43	9,62				

When SD's were not available, calculatable and could not be retrieved from the authors, we used the mean SD for that dataset (for example, HF dataset, pre-treatment SD). These SD's are written in red.

means data retrieved from figures in paper

***** means data retrieved from author when requested
