# Supplementary material for: More rTMS pulses or more sessions? The impact on treatment outcome for treatment resistant depression
Source: Acta Psychiatr Scand. 2024 Nov 21;151(4):485–505. doi: 10.1111/acps.13768 (PMC11884915; doi:10.1111/acps.13768)
Supplement: Supplementary file 2 — Data S2. Supporting Information. [file ACPS-151-485-s001.docx]

**Supplementary**

S2.0 Supplementary methods

S2.1 Search strategy

Herewith the full strategy of our literature search.

**A. MESH terms and free terms used for PubMed**

**# rTMS**

"Transcranial Magnetic Stimulation"[Mesh] OR "transcranial magnetic stimulation*"[tiab] OR “repetitive transcranial magnetic”[tiab] OR “rTMS”[tiab] OR “TMS”[tiab]

**# Depression**

"Depression"[Mesh] OR "Depressive Disorder"[Mesh] OR "Bipolar Disorder"[Mesh] OR "Cyclothymic Disorder"[Mesh] OR “depress*”[tiab] OR “dysthymi*”[tiab] OR “antidepress*”[tiab] OR “anti-depress*”[tiab] OR “mood disorder*”[tiab] OR “bipolar disorder*”[tiab] OR “cyclothymi*”[tiab]

**# Studytype**(“Randomized Controlled Trial”[pt] OR “Randomized Controlled Trials as Topic”[Mesh] OR “Random allocation” [Mesh] OR “Double-blind method”[Mesh] OR “Single-blind method”[Mesh] OR “Placebos”[Mesh] OR "Clinical Trials as Topic"[Mesh] OR “Clinical Trial”[pt] OR “Controlled Clinical Trial”[pt] OR "Pilot Projects"[Mesh] OR "Clinical Protocols"[Mesh:NoExp] OR "Clinical Trial Protocols as Topic"[Mesh] OR "Clinical Trial Protocol" [Publication Type] OR random[tiab] OR randomly[tiab] OR randomised[tiab] OR randomized[tiab] OR randomising[tiab] OR randomizing [tiab] OR placebo* OR ((singl*[tiab] OR doubl*[tiab] OR trebl*[tiab] OR tripl*[tiab]) AND (mask*[tiab] OR blind*[tiab] OR dumm*[tiab])) OR RCT[tiab] OR RCTs[tiab] OR “clinical trial*”[tiab] OR “controlled trial*”[tiab] OR “pilot project*”[tiab] OR “pilot stud*”[tiab] OR “trial protocol*”[tiab] OR “research protocol*”[tiab] OR “study protocol*”[tiab]) NOT (“Animals”[Mesh] NOT “Humans”[Mesh])

**B. Emtree terms and free terms used for Embase (Embase.com)**

**# rTMS**

'transcranial magnetic stimulation'/exp OR ‘transcranial magnetic stimulation*’:ab,ti,kw OR ‘repetitive transcranial magnetic’:ab,ti,kw OR ‘rTMS’:ab,ti,kw OR ‘TMS’:ab,ti,kw

**# Depression**

'depression'/exp OR ‘depress*’:ab,ti,kw OR ‘dysthymi*’:ab,ti,kw OR ‘antidepress*’:ab,ti,kw OR ‘anti-depress*’:ab,ti,kw OR ‘mood disorder*’:ab,ti,kw OR ‘bipolar disorder*’:ab,ti,kw OR ‘cyclothymi*’:ab,ti,kw

**# Studytype**

'clinical protocol'/de OR 'clinical trial'/de OR 'clinical trial topic'/de OR 'controlled clinical trial'/de OR 'controlled study'/de OR 'pilot study'/de OR 'randomized controlled trial'/de OR 'randomized controlled trial topic'/de OR 'randomization'/de OR 'double blind procedure'/exp OR 'single blind procedure'/exp OR 'placebo'/exp OR 'clinical trial protocol'/exp OR random:ab,ti,kw OR randomly:ab,ti,kw OR randomised:ab,ti,kw OR randomized:ab,ti,kw OR randomising:ab,ti,kw OR randomizing:ab,ti,kw OR placebo*:ab,ti,kw OR ((singl* OR doubl* OR trebl* OR tripl*) NEAR/3 (mask* OR blind* OR dumm*)):ab,ti,kw OR RCT*:ab,ti,kw OR ‘clinical trial*’:ab,ti,kw OR ‘controlled trial*’:ab,ti,kw OR ‘pilot project*’:ab,ti,kw OR ‘pilot stud*’:ab,ti,kw OR ‘trial protocol*’:ab,ti,kw OR ‘research protocol*’:ab,ti,kw OR ‘study protocol*’:ab,ti,kw

**# Publicatie type**

('article'/it OR 'article in press'/it OR 'conference paper'/it OR 'letter'/it OR 'note'/it OR 'review'/it OR 'short survey'/it)

**C. APA Thesaurus terms and free terms used for APA PsycInfo (EBSCO)**

**# rTMS**

DE "Transcranial Magnetic Stimulation" OR TI("transcranial magnetic stimulation*" OR “repetitive transcranial magnetic” OR “rTMS” OR “TMS”) OR AB("transcranial magnetic stimulation*" OR “repetitive transcranial magnetic” OR “rTMS” OR “TMS”) OR KW("transcranial magnetic stimulation*" OR “repetitive transcranial magnetic” OR “rTMS” OR “TMS”)

**# Depression**

DE "Anaclitic Depression" OR DE "Dysthymic Disorder" OR DE "Endogenous Depression" OR DE "Late Life Depression" OR DE "Postpartum Depression" OR DE "Reactive Depression" OR DE "Recurrent Depression" OR DE "Treatment Resistant Depression" OR DE "Atypical Depression" OR DE "Depression (Emotion)" OR DE "Bipolar Disorder" OR DE "Bipolar I Disorder" OR DE "Bipolar II Disorder" OR DE "Major Depression" OR DE "Cyclothymic Disorder" OR TI(“depress*” OR “dysthymi*” OR “antidepress*” OR “anti-depress*” OR “mood disorder*” OR “bipolar disorder*” OR “cyclothymi*”) OR AB(“depress*” OR “dysthymi*” OR “antidepress*” OR “anti-depress*” OR “mood disorder*” OR “bipolar disorder*” OR “cyclothymi*”) OR KW(“depress*” OR “dysthymi*” OR “antidepress*” OR “anti-depress*” OR “mood disorder*” OR “bipolar disorder*” OR “cyclothymi*”)

**# Studietype**

DE "Randomized Clinical Trials" OR DE "Clinical Trials" OR DE "Randomized Controlled Trials" OR DE "Placebo" OR TI(random OR randomly OR randomised OR randomized OR randomising OR randomizing OR placebo* OR ((singl* OR doubl* OR trebl* OR tripl*) W3 (mask* OR blind* OR dumm*)) OR RCT* OR “clinical trial*” OR “controlled trial*” OR “pilot project*” OR “pilot stud*” OR “trial protocol*” OR “research protocol*” OR “study protocol*”) OR AB (random OR randomly OR randomised OR randomized OR randomising OR randomizing OR placebo* OR ((singl* OR doubl* OR trebl* OR tripl*) W3 (mask* OR blind* OR dumm*)) OR RCT OR “clinical trial*” OR “controlled trial*” OR “pilot project*” OR “pilot stud*” OR “trial protocol*” OR “research protocol*” OR “study protocol*”) OR KW(random OR randomly OR randomised OR randomized OR randomising OR randomizing OR placebo* OR ((singl* OR doubl* OR trebl* OR tripl*) W3 (mask* OR blind* OR dumm*)) OR RCT OR “clinical trial*” OR “controlled trial*” OR “pilot project*” OR “pilot stud*” OR “trial protocol*” OR “research protocol*” OR “study protocol*”)

Narrow by Methodology: - clinical trial

**# Publicatie type**

Limit to academic journals

**D. Topic search (Title, abstracts and Keyword Plus) in Web of Science (Clarivate)**

**# rTMS**

TS=("transcranial magnetic stimulation*" OR “repetitive transcranial magnetic” OR “rTMS” OR “TMS”)

**# Depression**

TS=(“depress*” OR “dysthymi*” OR “antidepress*” OR “anti-depress*” OR “mood disorder*” OR “bipolar disorder*” OR “cyclothymi*”)

**# Studietype**

TS=(random OR randomly OR randomised OR randomized OR randomising OR randomizing OR placebo* OR ((singl* OR doubl* OR trebl* OR tripl*) NEAR/3 (mask* OR blind* OR dumm*)) OR RCT* OR “clinical trial*” OR “controlled trial*” OR “pilot project*” OR “pilot stud*” OR “trial protocol*” OR “research protocol*” OR “study protocol*”)

**# publicatie type**

Article or Review Article or Proceeding Paper or Letter or Early Access (Document Types)

S2.2 Formulas used for primary outcome

SMD was calculated using formula (4.26), derived from Borenstein et al.[1]:

$SMD= \frac{\left( M_{pre}-M_{post} \right)}{{SD}_{pre\_pooled}}$

Where *M* stands for mean depression rating score, *pre* for baseline and *post* for post-treatment measurement.

*SD_pre_pooled_* was calculated using formula (4.4), derived from Borenstein et al. [1]:

${SD}_{pre\_pooled}= \sqrt{\frac{\left( \left( n1-1 \right)* {SD}_{pre 1}^{2} \right)+\ldots\left( \left( nk-1 \right)* {SD}_{pre k}^{2} \right)}{(n1+\ldots nk-2)}}$

Where *n* stands for number of subjects in the *k* arm.

The variance of the SMD (*Vd*) was calculated following formula (4.28) [1]:

$$V_{d}=\left( \frac{1}{n}+ \frac{{SMD}^{2}}{2n} \right)*2\left( 1-r \right)$$

Here *n* is the number of pairs and *r* is the correlation coefficient between pairs of observation.

Formula used for the between groups differences, only included the RCTs with sham-rTMS as the control group [1]:

$${SMD}_{b}= \frac{\left( M_{preT}-M_{postT} \right)-\left( M_{preC}-M_{postC} \right)}{{SD}_{pre\_pooled}}$$

S3.0 Results


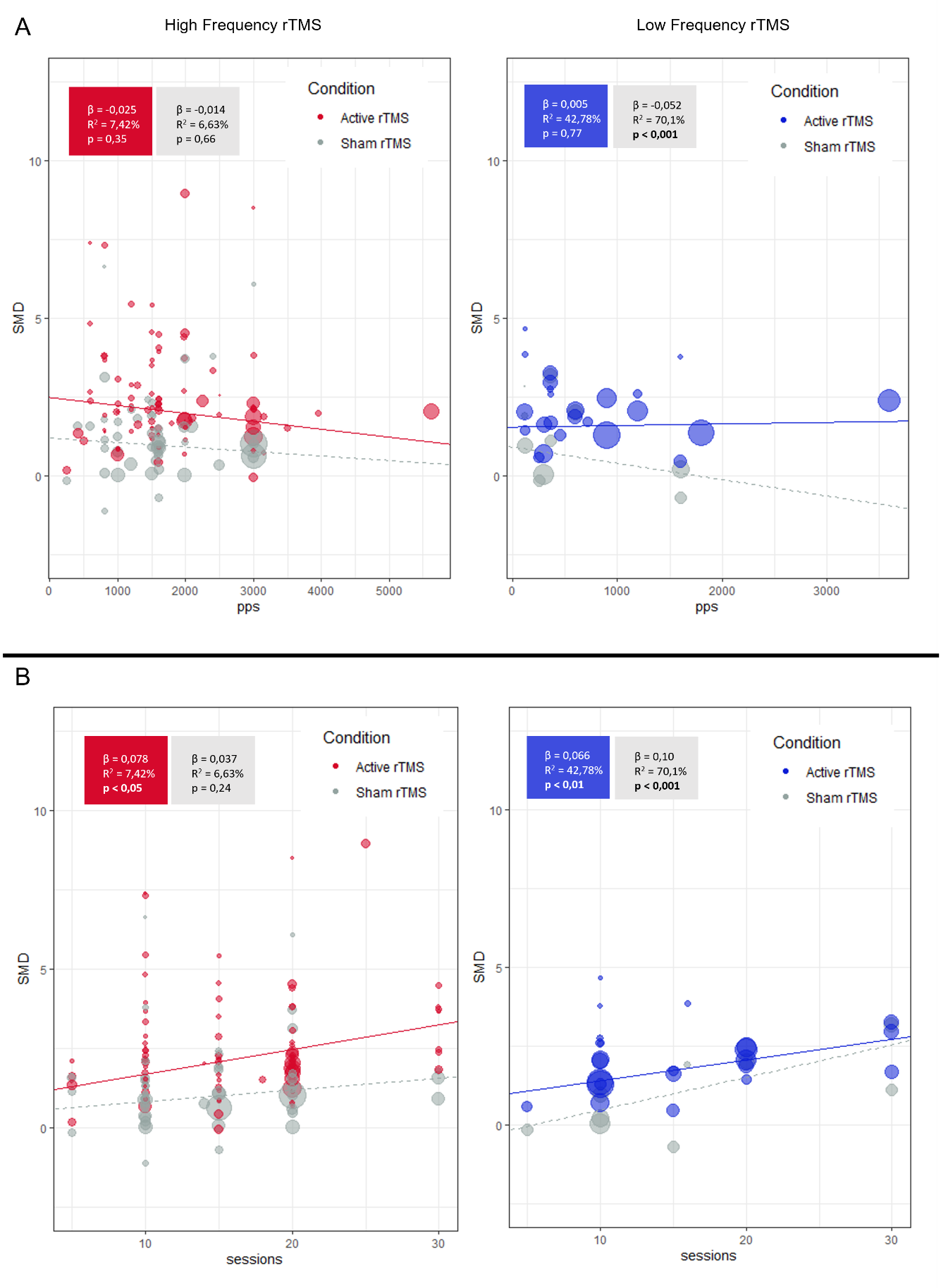


**Figure S1:** **Meta-regressions of the total amount of pulses or total number of sessions as independent variable and SMD as dependent variable.** A: meta-regression of HF-rTMS (red) en LF-rTMS (blue) studies between pulses/session and SMD, corrected for total number of sessions. B: meta-regression of HF-rTMS (red) en LF-rTMS (blue) studies between sessions and SMD, corrected for pulses/session. *SMD = standardized mean difference, HF = high-frequency, LF = low-frequency, rTMS = repetitive transcranial magnetic stimulation*

Subgroup effect size analyses


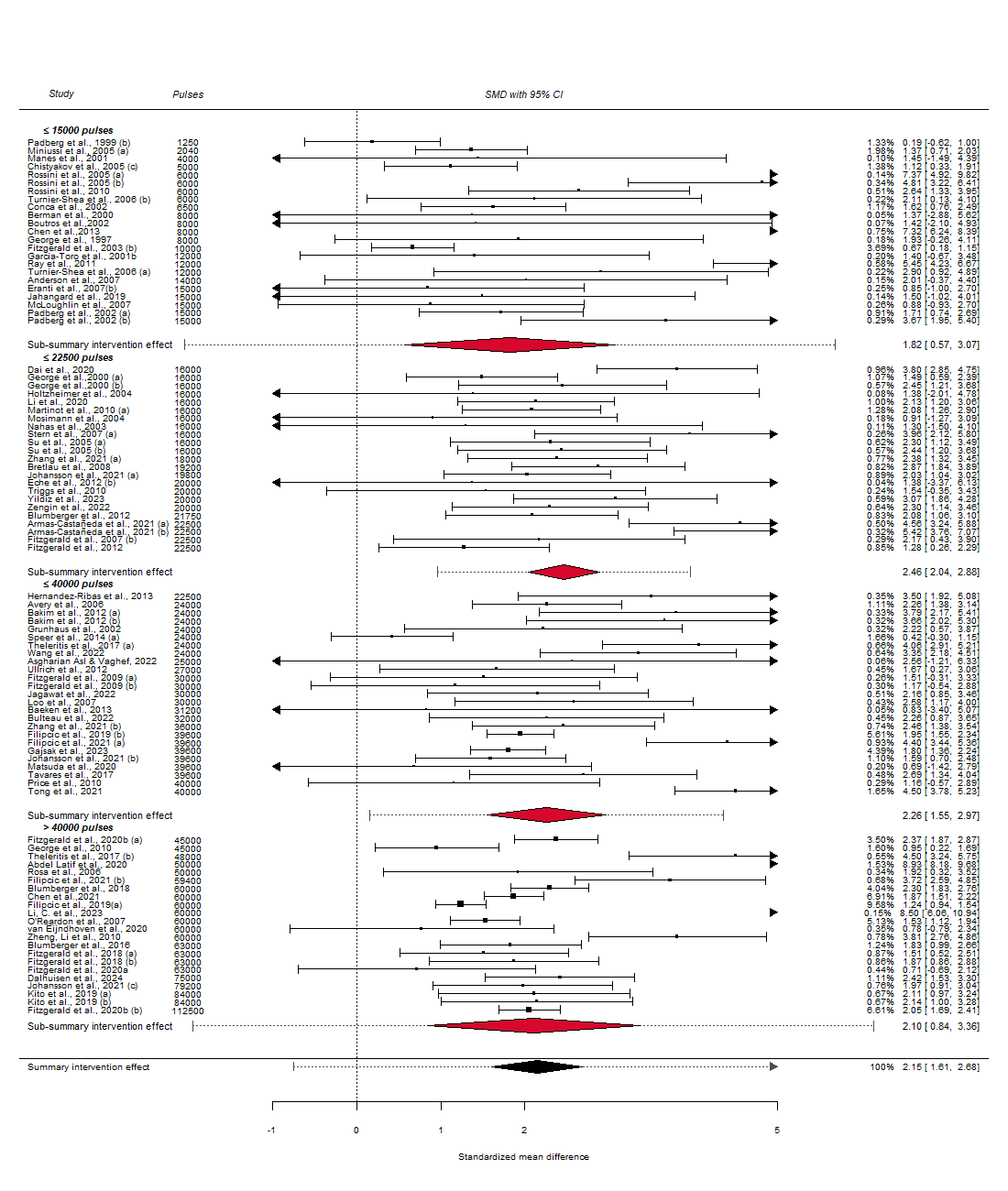


**Figure S2:** **Subgroup effect size analysis of HF-rTMS pulses.** Dataset of HF-rTMS studies is divided in quartiles based on the amount of rTMS pulses administered. Subgroup effect size analyses were done for every quartile. *SMD = standardized mean difference, HF = high-frequency, LF = low-frequency, rTMS = repetitive transcranial magnetic stimulation*


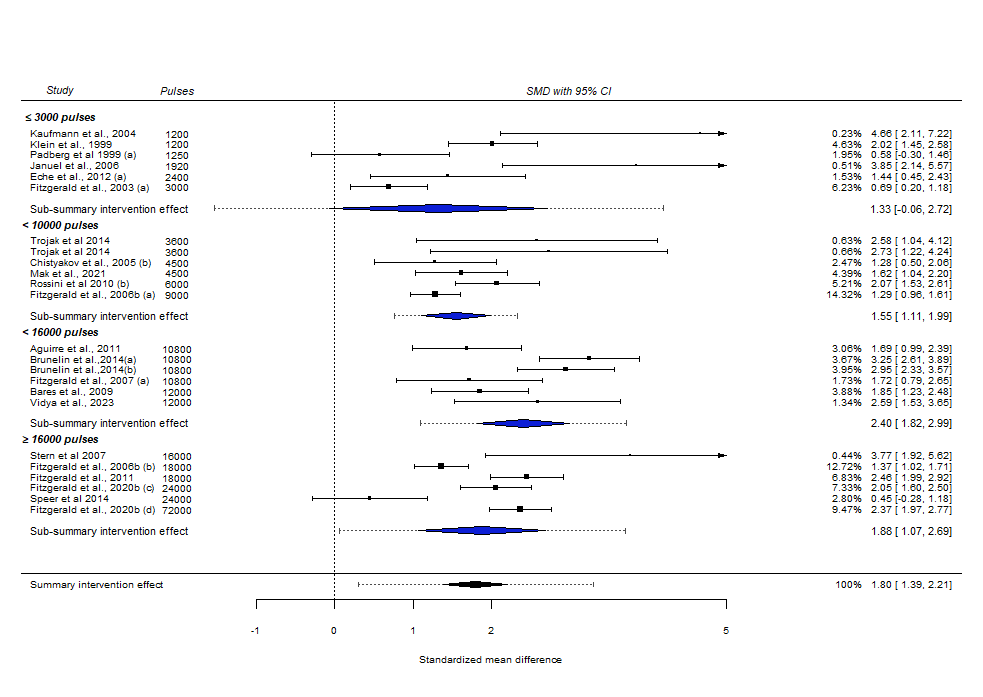


**Figure S3:** **Subgroup effect size analysis of LF-rTMS pulses.** Dataset of HF-rTMS studies is divided in quartiles based on the amount of rTMS pulses administered. Subgroup effect size analyses were done for every quartile. *SMD = standardized mean difference, HF = high-frequency, LF = low-frequency, rTMS = repetitive transcranial magnetic stimulation*


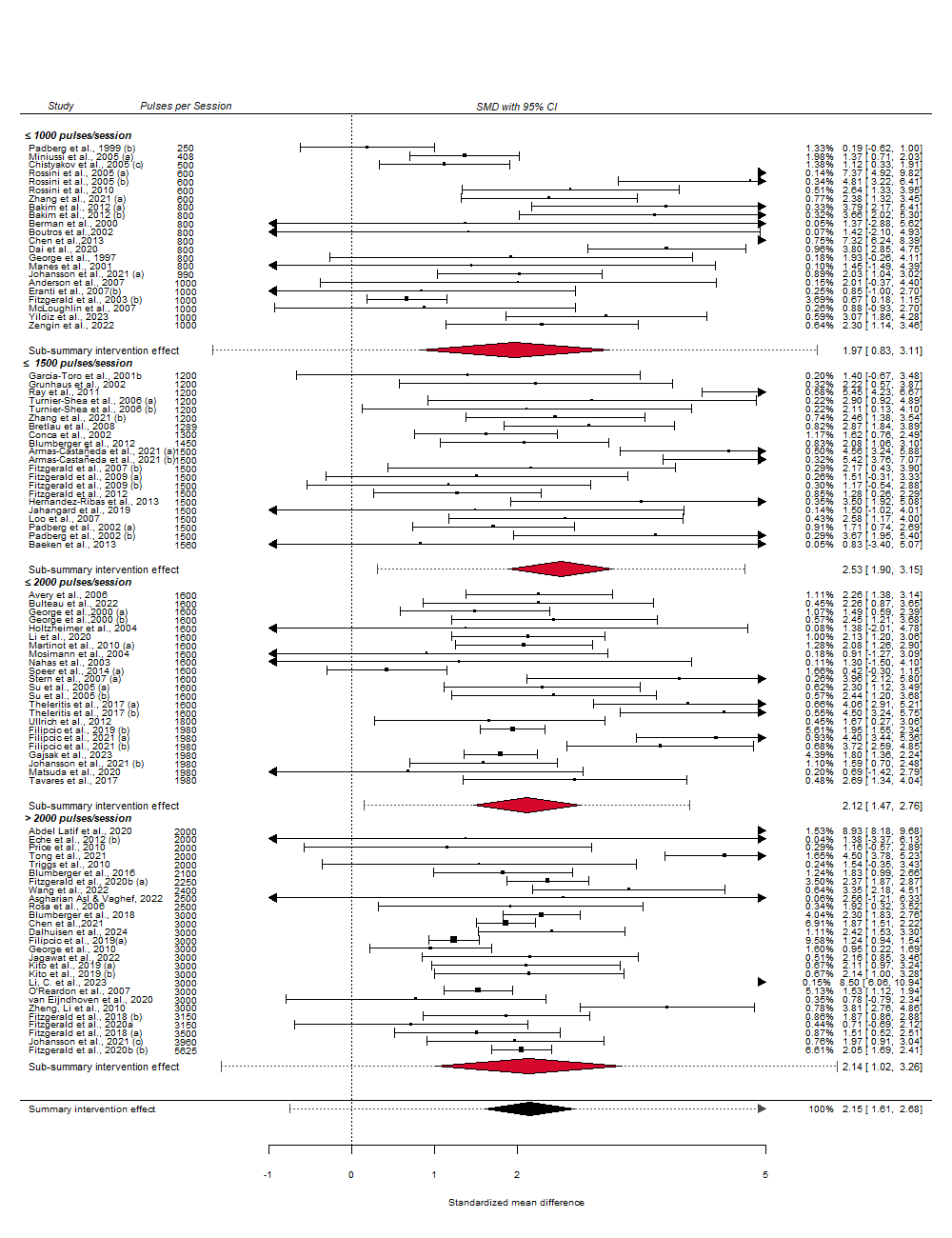


**Figure S4:** **Subgroup effect size analysis of HF-rTMS pulses/session.** Dataset of HF-rTMS studies is divided in quartiles based on the amount of pulses/session administered. Subgroup effect size analyses were done for every quartile. *SMD = standardized mean difference, HF = high-frequency, LF = low-frequency, rTMS = repetitive transcranial magnetic stimulation*


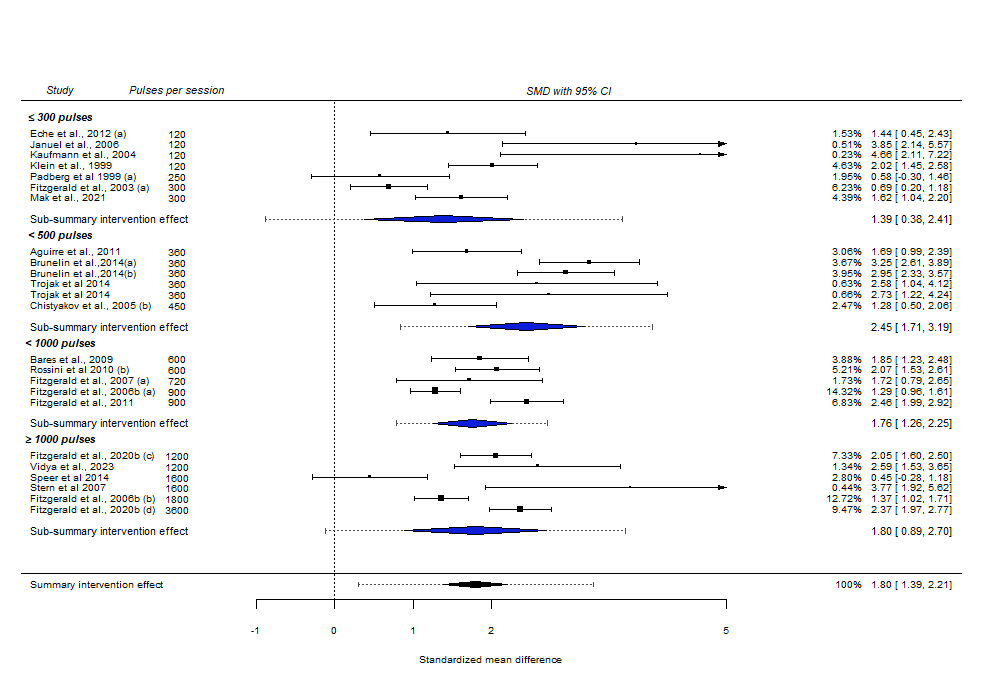


**Figure S5:** **Subgroup effect size analysis of LF-rTMS pulses/session.** Dataset of HF-rTMS studies is divided in quartiles based on the amount of pulses/session administered. Subgroup effect size analyses were done for every quartile. *SMD = standardized mean difference, HF = high-frequency, LF = low-frequency, rTMS = repetitive transcranial magnetic stimulation*


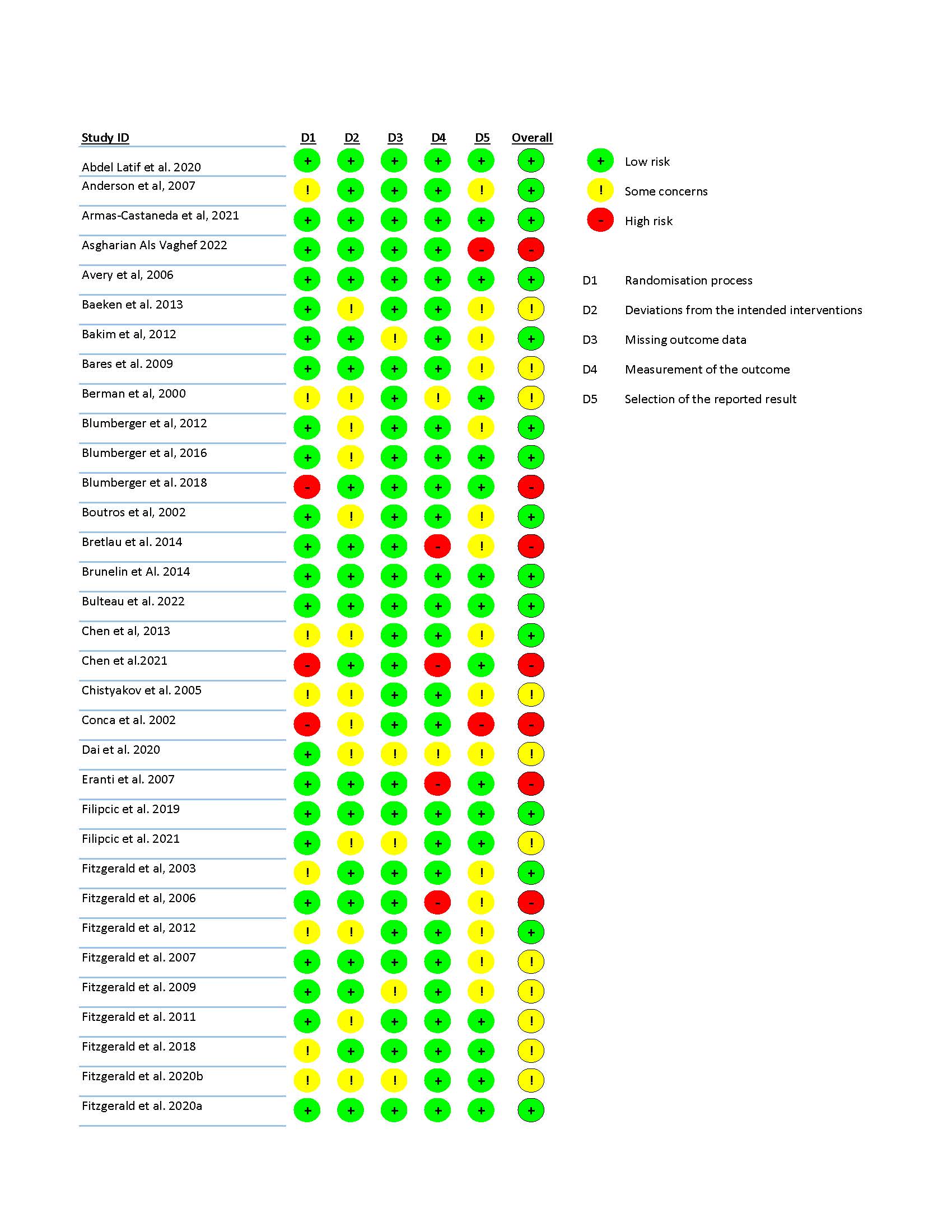
S3.1 Publication bias


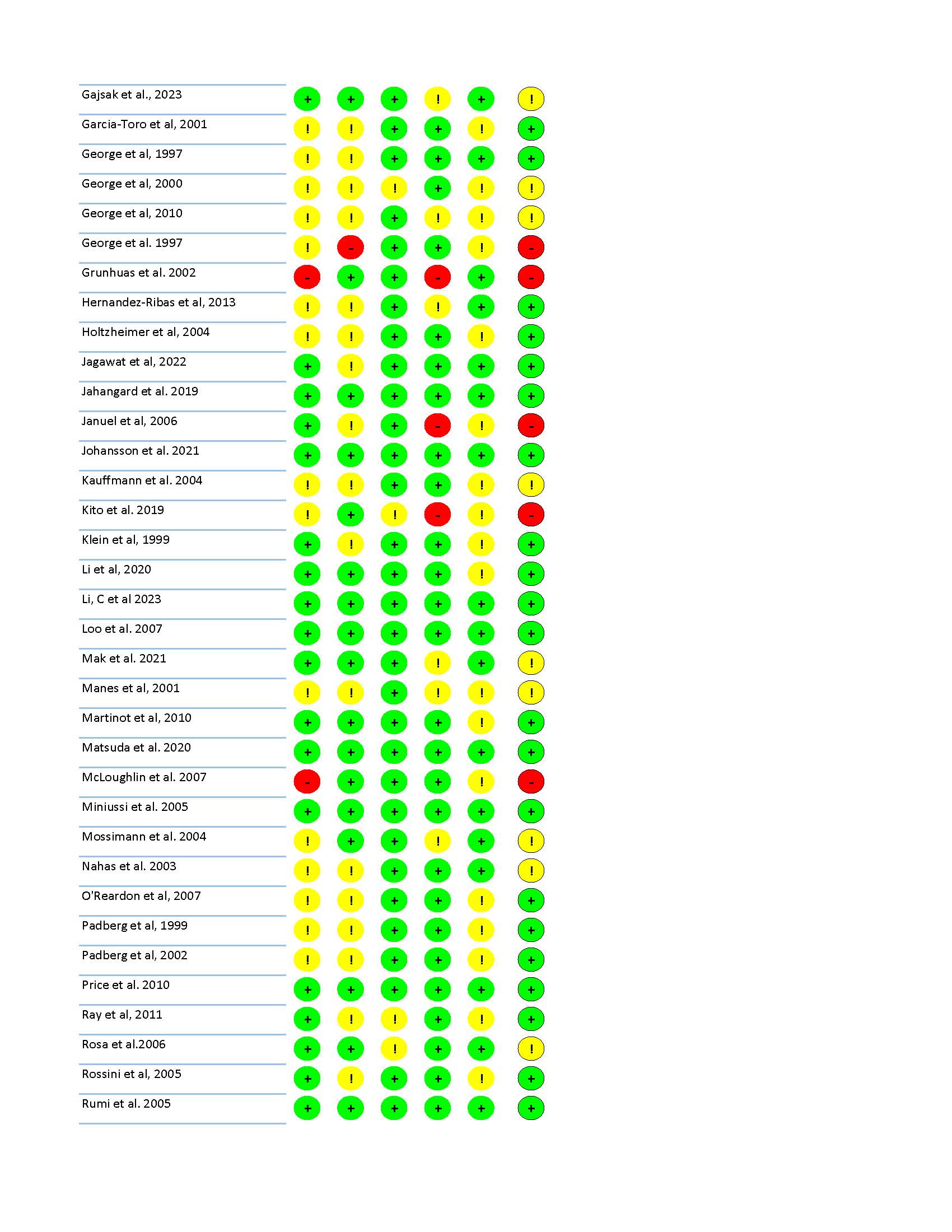


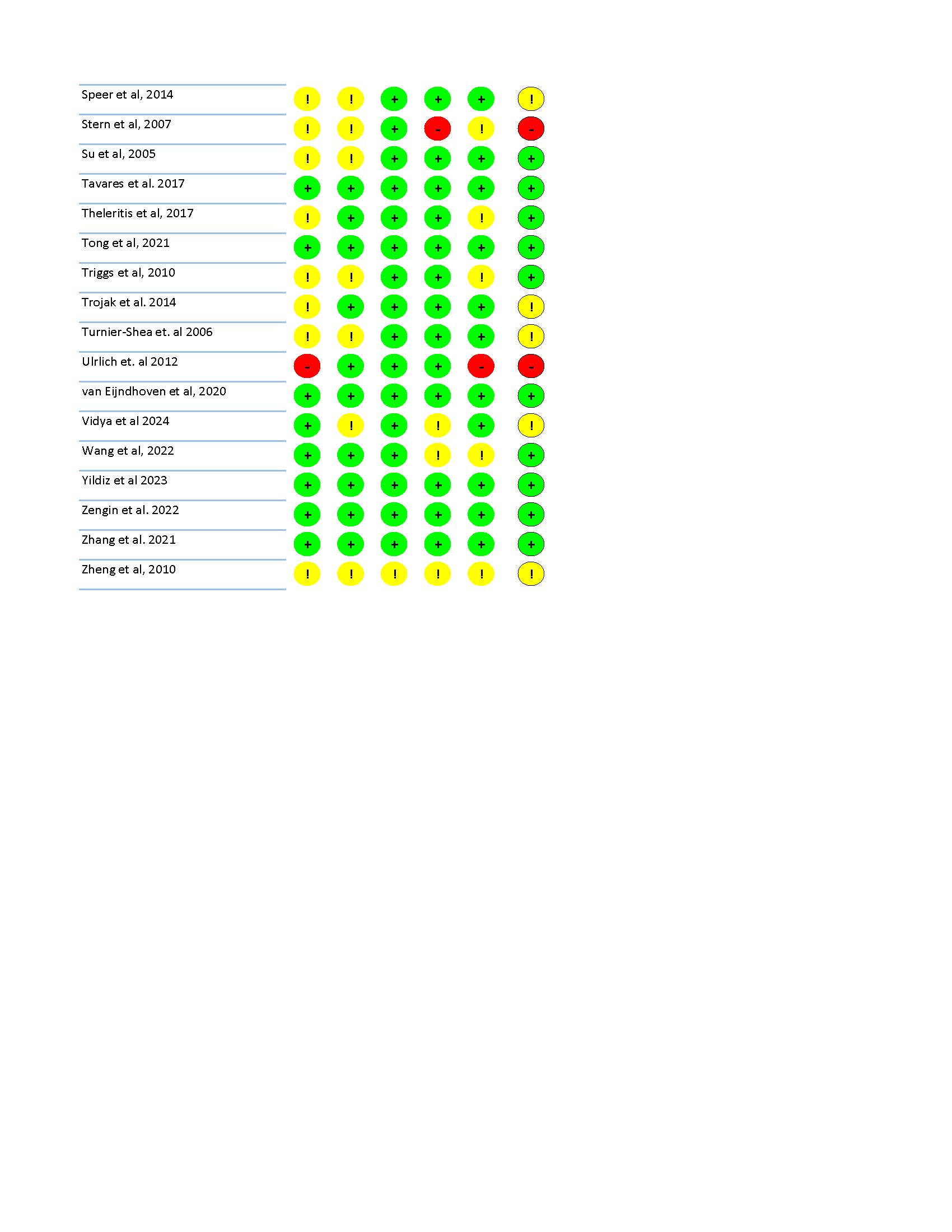


S3.2 Sensitivity analyses

**Table S1. Meta-analysis done for all of the included RCTs (only active rTMS arms of both HF and LF protocols), with sub-group effect size based on TMS device.**

| **Different TMS devices** | | | | | | | |
| --- | --- | --- | --- | --- | --- | --- | --- |
|  | | | | | | | |
|  | | | | | | | |
| **Device (n studies)** | **Effect (SE)** | **CI** | **τ** | **I^2^** | **H^2^** | **z** | **p** |
| *Brainsway (2)* | 2.11 (1.02) | 0.11 - 4.11 | 1.10 | 59.54% | 2.47 | 2.07 | **0.0387 *** |
| *Cadwell (6)* | 1.35 (0.33) | 0.71 - 1.99 | 0.65 | 66.35% | 2.97 | 4.12 | **< 0.0001***** |
| *MagPro (25)* | 2.00 (0.25) | 1.51 - 2.49 | 0.87 | 84.06% | 6.27 | 7.97 | **< 0.0001***** |
| *Magstim (42)* | 2.07 (0.38) | 1.32 - 2.82 | 1.67 | 93.33% | 14.99 | 5.40 | **< 0.0001***** |
| *Multi (6)* | 2.52 (0.46) | 1.62 - 3.41 | 0.73 | 79.53% | 4.88 | 5.50 | **< 0.0001***** |
| *Neurentics (5)* | 1.59 (0.53) | 0.55 - 2.64 | 0.74 | 77.45% | 4.43 | 2.98 | **0.0029 **** |

**Table S2. Meta-regression results without outlier (Fitzgerald et al., 2020b [2])**

| **A: Total number of sessions and total amount of pulses** | | | | | | | | |
| --- | --- | --- | --- | --- | --- | --- | --- | --- |
| **HF** |  | **β (SE)** | **τ** | **I^2^** | **H^2^** | **R^2^** | **z** | **p** |
| *Active* | *Sessions* | 0.1292 (0.0452) | 1.4072 | 89.50% | 9.52 | 8.61% | 2.8571 | **0.0043**** |
|  | *Pulses* | -0.0273 (0.0164) |  |  |  |  | -1.6624 | 0.0964 |
| *Sham* | *Sessions* | 0.0529  (0.0483) | 0.9397 | 90.88% | 10.97 | 6.54% | 1.0959 | 0.2731 |
|  | *Pulses* | -0.0096 (0.0201) |  |  |  |  | -0.4787 | 0.6321 |
| **LF** |  |  |  |  |  |  |  |  |
| *Active* | *Sessions* | 0.0657 (0.0232) | 0.5535 | 73.69% | 3.80 | 41.90% | 2.8333 | **0.0046 **** |
|  | *Pulses* | -0.0087 (0.0242) |  |  |  |  | -0.3608 | 0.7182 |
| *Sham* | *Sessions* | 0.1219 (0.0291) | 0.5747 | 71.72% | 3.54 | 72.90% | 4.1895 | **<.0001** *** |
|  | *Pulses* | -0.0495 (0.0350) |  |  |  |  | -1.4149 | 0.1571 |
|  |  |  |  |  |  |  |  |  |
|  |  |  |  |  |  |  |  |  |
| **B: Total number of sessions and pulses per session** | | | | | | | | |
| **HF** |  | **β (SE)** | **τ** | **I^2^** | **H^2^** | **R^2^** | **z** | **p** |
| *Active* | *Sessions* | 0.0964 (0.0340) | 1.4034 | 89.44% | 9.47 | 9.10% | 2.8395 | **0.0299 **** |
|  | *PPS* | -0.0005 (0.0003) |  |  |  |  | -1.6812 | 0.0927 |
| *Sham* | *Sessions* | 0.0368  (0.0312) | 0.9392 | 90.90% | 10.99 | 6.63% | 1.1806 | 0.2378 |
|  | *PPS* | -0.0001 (0.0003) |  |  |  |  | -0.4382 | 0.6613 |
| **LF** |  |  |  |  |  |  |  |  |
| *Active* | *Sessions* | 0.0611  (0.0219) | 0.5558 | 73.51% | 3.77 | 41.40% | 2.7862 | **0.0053 **** |
|  | *PPS* | -0.0106 (0.0339) |  |  |  |  | -0.3125 | 0.7546 |
| *Sham* | *Sessions* | 0.1030 (0.0291) | 0.6035 | 73.12% | 3.72 | 70.11% | 3.5425 | **0.0004** *** |
|  | *PPS* | -0.0515 (0.0450) |  |  |  |  | -1.1460 | 0.2518 |
|  |  |  |  |  |  |  |  |  |

**Table S3**. **Meta-regression results using two covariates with r = 0.2**

| **A: Total number of sessions and total amount of pulses** | | | | | | | | |
| --- | --- | --- | --- | --- | --- | --- | --- | --- |
| **HF** |  | **β (SE)** | **τ** | **I^2^** | **H^2^** | **R^2^** | **z** | **p** |
| *Active* | *Sessions* | 0.0921 (0.0506) | 1.5639 | 98.21% | 55.89 | 4.97% | 1.8218 | 0.0538 ^#^ |
|  | *Pulses* | -0.0125  (0.0150) |  |  |  |  | -0.8336 | 0.4045 |
| *Sham* | *Sessions* | 0.0529 (0.0624) | 1.2391 | 98.86% | 87.65 | 4.22% | 0.8484 | 0.3962 |
|  | *Pulses* | -0.0096 (0.0262) |  |  |  |  | -0.3670 | 0.7136 |
| **LF** |  |  |  |  |  |  |  |  |
| *Active* | *Sessions* | 0.0657 (0.0345) | 0.9042 | 97.39% | 38.45 | 10.45% | 1.9035 | 0.0570 ^#^ |
|  | *Pulses* | -0.0087  (0.0364) |  |  |  |  | 0.2437 | 0.8075 |
| *Sham* | *Sessions* | 0.1219 (0.0322) | 0.7172 | 95.18% | 20.75 | 63.40% | 3.7870 | **0.0002 ***** |
|  | *Pulses* | -0.0495  (0.0392) |  |  |  |  | -1.2619 | 0.2070 |
|  |  |  |  |  |  |  |  |  |
|  |  |  |  |  |  |  |  |  |
| **B: Total number of sessions and pulses per session** | | | | | | | | |
| **HF** |  | **β (SE)** | **τ** | **I^2^** | **H^2^** | **R^2^** | **z** | **p** |
| *Active* | *Sessions* | 0.0782 (0.0382) | 1.4835 | 97.99% | 49.65 | 6.17% | 2.0459 | **0.0408 *** |
|  | *PPS* | -0.025 (0.03) |  |  |  |  | -0.9378 | 0.3483 |
| *Sham* | *Sessions* | 0.0348 (0.0360) | 1.0965 | 98.59% | 70.69 | 2.12% | 0.9659 | 0.3341 |
|  | *PPS* | -0.014 (0.04) |  |  |  |  | -0.3783 | 0.7052 |
| **LF** |  |  |  |  |  |  |  |  |
| *Active* | *Sessions* | 0.0611 (0.0325) | 0.9131 | 97.40% | 38.43 | 8.68% | 1.8791 | 0.0602 |
|  | *PPS* | -0.005 (0.03) |  |  |  |  | 0.1785 | 0.8583 |
| *Sham* | *Sessions* | 0.1030 (0.0323) | 0.7484 | 95.44% | 21.92 | 60.15% | 3.1834 | **0.0015 **** |
|  | *PPS* | -0.0515 (0.05) |  |  |  |  | -1.0088 | 0.3131 |
|  |  |  |  |  |  |  |  |  |

**Table S4. Meta-regression results using two covariates with r = 0.8**

| **A: Total number of sessions + total amount of pulses** | | | | | | | | |
| --- | --- | --- | --- | --- | --- | --- | --- | --- |
| **HF** |  | **β (SE)** | **τ** | **I^2^** | **H^2^** | **R^2^** | **z** | **p** |
| *Active* | *Sessions* | 0.0939 (0.0471) | 1.4112 | 91.67% | 12.01 | 7.08% | 1.9934 | **0.0462 *** |
|  | *Pulses* | -0.013 (0.0000) |  |  |  |  | -0.9662 | 0.3339 |
| *Sham* | *Sessions* | 0.0514 (0.0470) | 0.8925 | 92.00% | 12.51 | 5.62% | 1.0933 | 0.2743 |
|  | *Pulses* | -0.009 (0.0000) |  |  |  |  | -0.5057 | 0.6130 |
| **LF** |  |  |  |  |  |  |  |  |
| *Active* | *Sessions* | 0.0650 (0.0231) | 0.5821 | 80.68% | 5.18 | 40.66% | 2.8113 | **0.0049 **** |
|  | *Pulses* | 0.004 (0.0000) |  |  |  |  | 0.3604 | 0.7185 |
| *Sham* | *Sessions* | 0.1219 (0.0294) | 0.5999 | 77.55% | 4.45 | 71.22% | 4.1514 | **<.0001 ***** |
|  | *Pulses* | -0.05 (0.0000) |  |  |  |  | -1.3972 | 0.1624 |
|  |  |  |  |  |  |  |  |  |
|  |  |  |  |  |  |  |  |  |
| **B: Total number of sessions + pulses per session** | | | | | | | | |
| **HF** |  | **β (SE)** | **τ** | **I^2^** | **H^2^** | **R^2^** | **z** | **p** |
| *Active* | *Sessions* | 0.0782 (0.0382) | 1.4104 | 91.66% | 11.99 | 7.18% | 2.1056 | **0.0352 *** |
|  | *PPS* | -0.025 (0.03) |  |  |  |  | -0.9783 | 0.3279 |
| *Sham* | *Sessions* | 0.0348 (0.0360) | 0.8981 | 92.12% | 12.69 | 4.43% | 1.1546 | 0.2482 |
|  | *PPS* | -0.014 (0.04) |  |  |  |  | -0.4576 | 0.6472 |
| **LF** |  |  |  |  |  |  |  |  |
| *Active* | *Sessions* | 0.0611 (0.0325) | 0.5827 | 80.72% | 5.19 | 40.53% | 2.9722 | **0.0030 **** |
|  | *PPS* | -0.005 (0.03) |  |  |  |  | 0.2635 | 0.7922 |
| *Sham* | *Sessions* | 0.1030 (0.0323) | 0.6291 | 78.70% | 4.70 | 68.34% | 3.5033 | **0.0005 ***** |
|  | *PPS* | -0.0515 (0.0005) |  |  |  |  | -1.1272 | 0.2597 |
|  |  |  |  |  |  |  |  |  |

**References**

[1] Borenstein M, Hedges LV, Higgins JP, Rothstein HR. Introduction to meta-analysis*.* John Wiley & Sons; 2021.

[2] Fitzgerald PB, Hoy KE, Reynolds J, Singh A, Gunewardene R, Slack C, et al. A pragmatic randomized controlled trial exploring the relationship between pulse number and response to repetitive transcranial magnetic stimulation treatment in depression. Brain Stimul 2020;13(1):145-52.
